# Opportunistic Feeding Strategy for the Earliest Old World Hypsodont Equids: Evidence from Stable Isotope and Dental Wear Proxies

**DOI:** 10.1371/journal.pone.0074463

**Published:** 2013-09-11

**Authors:** Thomas Tütken, Thomas M. Kaiser, Torsten Vennemann, Gildas Merceron

**Affiliations:** 1 Steinmann-Institut für Geologie, Mineralogie und Paläontologie, University of Bonn, Bonn, Germany; 2 University Hamburg, Biozentrum Grindel, Hamburg, Germany; 3 Institut de Géochimie, Université de Lausanne, Lausanne, Switzerland; 4 iPHEP UMR 7262 CNRS, Université de Poitiers, Poitiers, France; University of Arkansas, United States of America

## Abstract

**Background:**

The equid *Hippotherium primigenium*, with moderately hypsodont cheek teeth, rapidly dispersed through Eurasia in the early late Miocene. This dispersal of hipparions into the Old World represents a major faunal event during the Neogene. The reasons for this fast dispersal of *H. primigenium* within Europe are still unclear. Based on its hypsodonty, a high specialization in grazing is assumed although the feeding ecology of the earliest European hipparionines within a pure C_3_ plant ecosystem remains to be investigated.

**Methodology/Principal Findings:**

A multi-proxy approach, combining carbon and oxygen isotopes from enamel as well as dental meso- and microwear analyses of cheek teeth, was used to characterize the diet of the earliest European *H. primigenium* populations from four early Late Miocene localities in Germany (Eppelsheim, Höwenegg), Switzerland (Charmoille), and France (Soblay). Enamel δ^13^C values indicate a pure C_3_ plant diet with small (<1.4‰) seasonal variations for all four *H. primigenium* populations. Dental wear and carbon isotope compositions are compatible with dietary differences. Except for the Höwenegg hipparionines, dental microwear data indicate a browse-dominated diet. By contrast, the tooth mesowear patterns of all populations range from low to high abrasion suggesting a wide spectrum of food resources.

**Conclusions/Significance:**

Combined dental wear and stable isotope analysis enables refined palaeodietary reconstructions in C_3_ ecosystems. Different *H. primigenium* populations in Europe had a large spectrum of feeding habits with a high browsing component. The combination of specialized phenotypes such as hypsodont cheek teeth with a wide spectrum of diet illustrates a new example of the Liem’s paradox. This dietary flexibility associated with the capability to exploit abrasive food such as grasses probably contributed to the rapid dispersal of hipparionines from North America into Eurasia and the fast replacement of the brachydont equid *Anchitherium* by the hypsodont *H. primigenium* in Europe.

## Introduction

The radiation of tridactyl equids took place from the Middle Miocene onwards in North America, a few million years before their dispersal throughout Old World continents [1], [2]. This is one of the most popular textbook examples of evolution taught in palaeontology [3]. The expansion of grasslands in North America has initially been considered *the* environmental factor driving the evolution of high-crowned molars (hypsodonty) as an adaptation to abrasive diets (i.e. silica-rich grasses) among Miocene hipparionines, as well as many other grazing mammals [2], [4]. Abrasive material responsible for most of the tooth wear in large mammalian herbivores may have been plant silica (phytoliths) and/or mineral dust (grit) on the vegetation. The importance of grit versus phytoliths as abrasives for dental wear is, however, still controversial [5], [6], [7], [8], [9]. Open, arid (grassland) habitats clearly contain more abrasive food compared to more humid, forested habitats. Hypsodonty is thus considered as an adaptation to a more abrasive diet from grasslands because grasses contain more abrasive biogenic silica in the form of phytoliths (and/or grit) compared to leaves [10], [11], [12]. Recent studies question the causal link between grasslands and hypsodonty [9] and suggest a much more complex interplay of various factors [13], [14]. Indeed, based on phytolith analyses, Strömberg [14] conclude that grasslands were already more widespread in the Early Miocene prior to the increase of hypsodonty among many mammal taxa. Moreover, previous investigations suggest various ecological habits among Mio-Pliocene hypsodont hipparionines from North America, covering the entire spectrum between grazing and browsing [12], [15]. Thus characterizations of ungulate palaeo-diets solely based on crown height often do not reflect the full dietary breadth of a taxon. Selective pressures for crown height may have been weak in North American Equidae during prolonged periods of their evolution [12]. However, a stronger selection for the evolution of high-crowned dentitions occurred during the Early Miocene shortly before the first appearance of Equinae, the horse subfamily in which hypsodonty evolved [12] and that later migrated to Europe.

Neogene Old World equids are all derived from a North American common ancestor that dispersed into Eurasia across Beringia during times of glacio-eustatic sea level fall [2]. The arrival and subsequent radiation of hypsodont hipparionine horses of the *Cormohipparion* clade at the base of the Late Miocene is one of the most important dispersal events of the Eurasian Neogene. The first occurrence of high-crowned tridactyl hipparionine horses in western Eurasia (‘*Hipparion*’ Datum) has recently been pushed back to the early Late Miocene (MN 9, early Vallesian, ∼11.2 Ma) with discovery of *Hippotherium primigenium* in Atzelsdorf, Austria [16]. From this locality, isolated hipparionine molars were found in fluvio-lacustrine deposits in the palaeo-Danube delta, sandwiched between marine strata of two transgressions of Lake Pannon; the upper transgression being dated to between 11.2 and 11.1 Ma [16], [17]. The arrival of hipparionine horses in the eastern Mediterranean region at around 12–11 Ma was traditionally thought to mark the simultaneous westward expansion of savanna vegetation across the Old World. However, in Anatolia open landscapes with C_3_ grasslands were actually widespread from the Middle Miocene, prior the first occurrence of hipparionines in the Old World [18]. Indeed, C_4_ monocotyledons did not radiate across Western Eurasia during the Neogene [19], [20].

This study aims to investigate the feeding ecology of the earliest hipparionines from Europe within a pure C_3_ plant ecosystem in order to better understand one of the most significant faunal events during the Tertiary, the dispersal of hipparions into the Old World. Two scenarios can be considered: (1) hipparionines, because of their hypsodont cheek teeth, exploited grassy abrasive vegetation in open landscapes to spread into the Old World or, (2) their dispersal illustrates a new example of the Liem’s paradox [21], [22], inferring that specialized phenotypes (in this case hypsodont cheek teeth) enabled the species to occupy a wide ecological niche (a mixed-feeding or even browsing trait). These two hypotheses are addressed by analyzing combined dental wear (micro−/mesowear) and stable isotope (C and O) analyses of teeth from four populations of hipparionines that are amongst the earliest hipparionines in Europe.

The four localities, Eppelsheim (EP), Höwenegg (HO), Charmoille (CH), and Soblay (SOB), belong to the Late Miocene (MN9–MN10). The first two sites are situated in southern Germany, Charmoille is located in Switzerland and Soblay near Lyon in eastern France. In the latter locality two hipparionine taxa may have co-habited [23]. However, the large equid *Hippotherium primigenium* is always dominant in the faunal assemblage. Bernor et al. [24] thus assemble most of the Vallesian large hipparionines from Europe into a taxonomic unit defined as the “*Hippotherium primigenium*” complex.

Four different dietary proxies are examined: molar mesowear and microwear patterns in combination with carbon and oxygen isotope compositions of enamel carbonate of the early European *H. primigenium*. Stable isotope compositions of tooth enamel combined with dental micro- and mesowear analyses provide complementary information regarding dietary intake, habitat, and niche partitioning. These methods evaluate fundamentally different chemical (isotopes) and mechanical (dental wear) food properties. Although these dietary proxies are not completely taxon-independent due to some influences of the animals (digestive) physiology and masticatory food processing, this multi-proxy approach allows a refined dietary reconstruction for hypsodont horses. It contributes to a better understanding of the dietary flexibility and will thus help us better understand the successful rapid dispersal of *H. primigenium* into the Old World. Dental wear analysis will help to resolve the grazer-browser dichotomy while carbon and oxygen isotope analyses will yield complementary information regarding habitat and food properties.

### Fossil Sites with Early Hippotherium Analyzed in this Study

**Figure 1:**
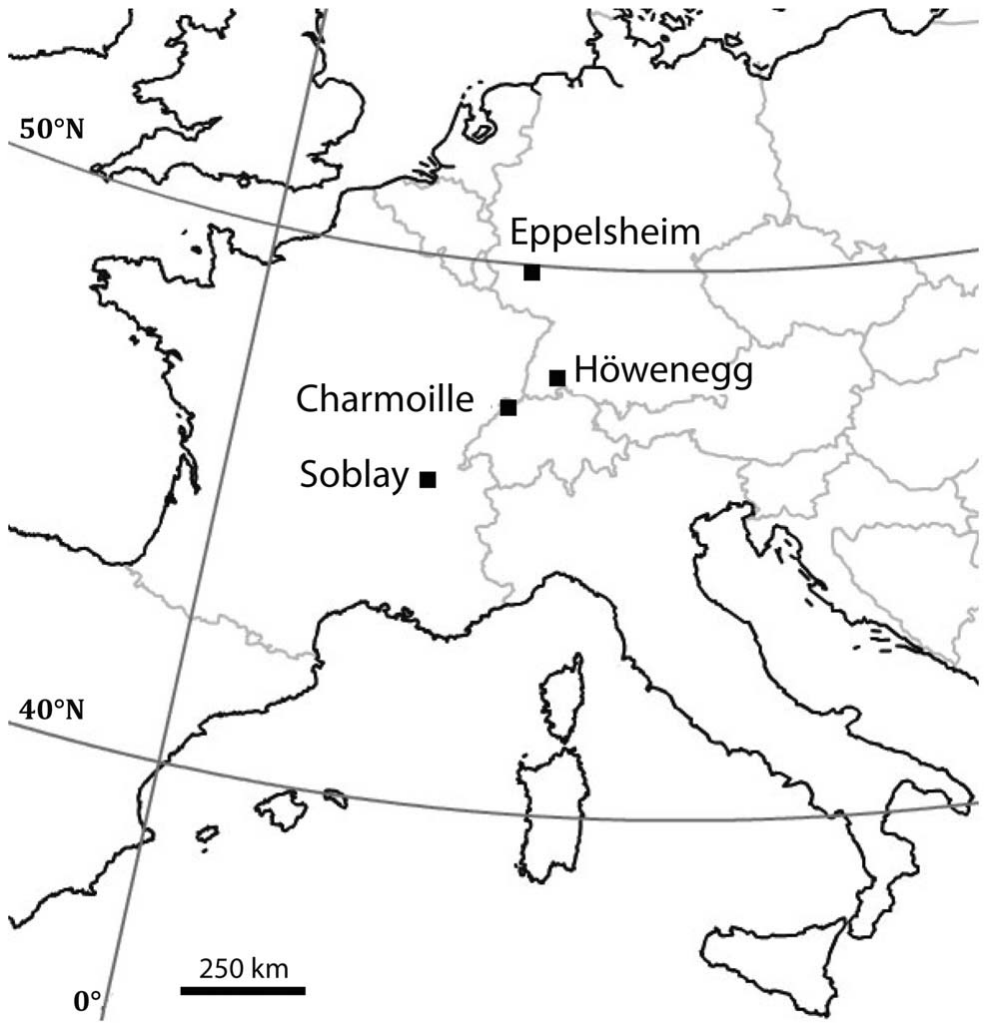
Geographic location of the investigated Late Miocene sampling sites.

The Eppelsheim locality (EP) belongs to the oldest deposits of the Miocene Rhine River, exposed at many places in the Rhine-Hesse area of Germany (Fig. 1). These fluvial sediments of the Eppelsheim Formation [25], [26], traditionally known as “Dinotheriensande”, have yielded many localities mainly placed stratigraphically within the Vallesian Land Mammal Age in the lower part of MN 9, the age of which is approximately 11.5 to 9.5 Ma [27], [28], [29]. However, in addition to Late Miocene (Vallesian) taxa the Dinotheriensand Fauna also contains early and late Middle Miocene mammal faunas [30]. Here, the focus is on Eppelsheim, situated 30 km south of the city of Mainz, which has provided the richest assemblage of mammalian remains among the Dinotheriensande complex [31], [32]. In addition, this assemblage contains the type-species sample of *Hippotherium primigenium* (MEYER, 1829), which is presently housed at the Forschungsinstitut Senckenberg (Frankfurt).

The Höwenegg locality (HO), located in southern Germany (Fig. 1), belongs to the lacustrine deposits of the Höwenegg-Formation, a sequence of light grey marl layers alternating with reddish-brown layers of tuffaceous mudflows. The Höwenegg-Formation was deposited in a lake that formed following the eruption of hornblende-bearing pyroclastics during the Late Miocene. The Höwenegg-Formation was dated to 10.3±0.19 Ma by a single hornblende crystal using ^40^Ar/^39^Ar from the hornblende tuff of the Höwenegg sequence [33], [34]. The early MN 9 Höwenegg locality is important due to this radiometric date for the ‘*Hipparion*’ Datum and because it contains completely articulated skeletons of *H. primigenium*.

The Late Miocene deposits in Soblay (SOB, Ain, France, Fig. 1) are composed of a several meter-thick sequence of lignites alternating with marls, which overly Upper Jurassic calcareous marine sediments. All fossil remains belong to the second lignite unit, which contains a rich mammalian fauna including 67 species of large and small mammals [23]. Based on the latter, the fauna is biostratigraphically dated to MN 10.

The Charmoille site (CH) is a sand pit close to the small town of Charmoille in the canton of Jura in northern Switzerland (Fig. 1). The faunal assemblage of Charmoille suggests that the locality belongs to the MN9 [35]. Indeed, the presence of *Hippotherium primigenium* together with two rhinocerotids *Aceratherium* cf. *incisivum* and *Dicerorhinus sansaniensis* support an early Vallesian age [36]. (See Text S1 for more detailed site and palaeoenvironmental descriptions).

### Combined Stable Isotope and Dental Wear Analyses - a Multi-proxy Dietary Approach

Hypsodont horse teeth grow over a period of several years and record in their enamel dietary, climatic and environmental information. Teeth of modern and fossil hypsodont horses have enamel growth rates of 35 to 40 mm/year and total enamel mineralization takes about 1 to 2.8 years depending on tooth type (molars usually take 1 to 1.5 years) [37], [38]. Similar rates of tooth mineralization can be assumed for the slightly less hypsodont Miocene *Hippotherium primigenium*. Due to a short turnover time of body water (about 14 days [37]) and its dissolved inorganic carbon pool, the carbon and oxygen isotopic composition of incrementally growing teeth of hypsodont herbivores record a time-series of the isotopic composition of dietary intake and seasonality [37], [39], [40], [41], [42].

### Stable Carbon Isotopes

The carbon isotope composition (δ^13^C) of animal tissues reflects that of the ingested food [43], [44] and is mainly related to the proportions of isotopically distinct C_3_ and C_4_ plants from dietary intake [44], [45]. C_3_ plants, which include trees, most shrubs and many cold-season, temperate latitudes and high altitude grasses, discriminate strongly against the heavy ^13^C isotope and therefore have lower δ^13^C values than C_4_ plants, ranging from –34 to –22‰, with an average of –27‰±3 [46]. C_4_ plants include mostly warm-season grasses that discriminate less against the heavy ^13^C isotope and therefore have higher, less negative δ^13^C values of between –17 and –9‰, with an average of –13‰±2 [46]. Carbon ingested with diet and incorporated into the structural carbonate of the enamel apatite is enriched in ^13^C by about +14‰ relative to the plants ingested by large non-ruminant herbivorous mammals such as horses, depending on the digestive physiology and rate of methane production of the animal [47], [48]. Therefore, skeletal apatite of extant animals with a pure C_3_ plant diet have average δ^13^C values of about –13‰ while animals feeding on a pure C_4_ plant diet have an average δ^13^C value of about +1‰ [49], [50]. The large C_4_ grass-dominated grassland ecosystems of savannas known today first evolved globally in the late Miocene [20], [51]. However, this is in contrast with data from Europe where C_3_ plants dominated the vegetation during the Neogene, as indicated by the observed low δ^13^C values in fossil mammal teeth of herbivores [19], [52]. In a C_3_ plant-dominated ecosystem C_3_ grazers and C_3_ browsers cannot be easily distinguished by carbon isotope analysis of most tissues. However, the enamel carbon isotopic composition in C_3_ ecosystems enables the niche partitioning and habitat differences of herbivorous mammals to be determined [53], [54], [55], [56]. This is due to the variability of δ^13^C values even within the C_3_ plant groups, due to variations in light and water availability as well as position in the forest canopy or habitat temperature, depending on latitude and altitude [57], [58], [59].

### Stable Oxygen Isotopes

Mammalian bioapatite records body water and hence ingested meteoric water δ^18^O values. The body water δ^18^O value of obligate drinkers, such as most large mammals including equids, is linearly related to that of the drinking water [60], [61], [62]. The δ^18^O_H2O_ values of meteoric water vary within ecosystems due to changes in air temperature and/or amount of precipitation or evaporation [63], [64]. These meteoric water δ^18^O_H2O_ differences can be used to infer climatic conditions such as ambient air temperature and aridity as well as animal drinking behaviour [60], [61], [65], [66]. Herbivorous mammals derive their water from three sources: (1) surface water, (2) water from food, and (3) metabolic water from food processing, specifically during oxidation of carbohydrates [61], [67]. Thus physiological, environmental, and behavioural factors can influence enamel δ^18^O values, particularly the water dependency of the animal [61], [65], [68]. Enamel δ^18^O values of obligate drinking mammals are dependent on meteoric δ^18^O values whereas drought-tolerant animals usually have less negative δ^18^O values because they obtain proportionally more water from ^18^O-enriched food sources such as leaves, fruits or seeds [61], [69]. Browsing taxa that ingest a higher proportion of ^18^O-enriched water with their food often have higher relative ^18^O values compared to sympatric grazing taxa [68], [70]. Therefore, δ^18^O values allow us to draw inferences regarding habitat properties, feeding ecology, drinking behaviour and humidity [65], [69], [70], [71]. Seasonality is recorded as δ^18^O amplitude changes in high crowned horse teeth which allows the evaluation of climatic changes [37], [72], [73]. Combined oxygen and carbon isotope analyses on hypsodont teeth also allow for the reconstruction of seasonal changes of ingested water and diet [42], [72], [74].

### Dental Meso- and Microwear

Mechanical abrasion caused by dietary intake leaves its traces at the enamel surface [7], [75], [76]. Thus micro- and mesowear investigations of fossil herbivore teeth provide additional information about the type of consumed plant material and the palaeohabitat [77], [78], [79], [80], [81], [82], [83], [84], [85], [86].

Dental facet development on the molar surfaces of living herbivorous ungulates appears to be strongly tied to their feeding styles [86]. These mesowear patterns reflect the long-term (several months to years) diet and can be used to infer broad dietary habits in extinct ungulates [12], [82], [85]. Dental mesowear reflects the degree of attritional and abrasive wear on the molar occlusal surface. Attritional wear is due to tooth-on-tooth contact and results in high crown relief and sharp cusp apices. Abrasive wear, on the other hand, is due to the alteration of enamel tissue by food items during mastication. In contrast to attritional wear, abrasion obliterates dental facets resulting in lower crown relief and rounder apices on cheek teeth [86].

Dental microwear patterns reflect a short-term (a few days to weeks) dietary signal of the physical properties of the last food items consumed by an individual [7], [76], [79]. Ungulates, whose main food resources are graminoids (including grasses and grass-like plants, such as sedges), have higher densities of scratches (elongated microwear scars) than pits (short and round microwear scars) [87]. This intense scratching observed in grazing ungulates is due to the abrasiveness of monocotyledons. The cell walls of monocotyledons contain a high concentration of silica phytoliths [88], [89], the abrasiveness of which is considered as an adaptive response to herbivory [90]. In contrast to monocotyledons, dicotyledons have fewer silica phytoliths, such that browsing ungulates have a lower ratio of scratches to pits compared to grazers. Beyond the grazer/browser dichotomy, the dental microwear method has been used to detect more subtle feeding preferences such as browsers whose diets contain fruits and seeds, or mixed-feeders that switch from grazing to browsing on a daily basis [91], [92].

It is worth noting here that two recent studies based on scratch tests and hardness measurements conclude that silica phytoliths do not appear to influence the enamel enough to scratch it, but identify grit as *the* major factor driving dental abrasion [8], [9]. In contrast, empirical dental microwear data from mammals indicates that variations in food properties play a major role in controlling the microwear patterns. These studies include investigations of mammal populations from the polyspecific scale [93], [94] to the monospecific scale [79], [95], as well as from controlled feeding studies [7], [76] and abrasion experiments with teeth mounted on a tribometer [75]. For example, reindeer feeding on ground lichens in tundra settings or camelids browsing in arid and dusty habitats differ from other ungulates by a higher amount of pits on the enamel surface [5], [91]. Thus ingestion of mineral particles does not cause a “grazing” dental microwear pattern with many scratches for these browsing species, demonstrating that the grit contribution to dental wear is minor compared to abrasive plant matter.

## Results

### Carbon and Oxygen Isotope Compositions of the Hipparion Teeth

**Figure 2:**
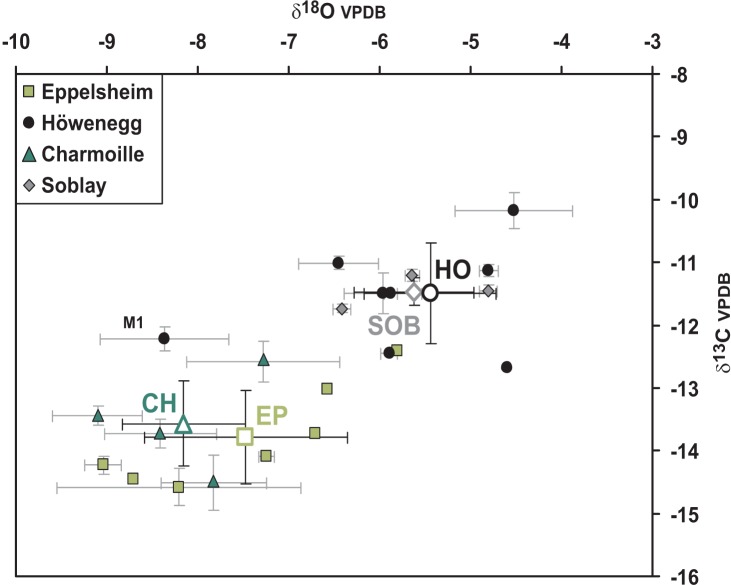
δ^13^C and δ^18^O values of all 21 *H. primigenium* teeth from the four different localities Eppelsheim (EP), Höwenegg (HO), Charmoille (CH) and Soblay (SOB). Note that the M1 from HO was excluded for the calculation of the HO mean value. For serially sampled teeth, average values and standard deviations are given. Mean values and standard deviations of each locality are plotted as open symbols.

Average δ^13^C values of the carbonate in the enamel bioapatite from 22 *Hippotherium* teeth of the four Late Miocene localities EP, CH, HO, and SOB range from −14.6 to −10.2‰ (Table 1). These values are typical for a pure C_3_ plant diet in all analyzed *Hippotherium* individuals. However, there are significant inter-site differences: EP and CH specimens have more negative mean δ^13^C values ranging from −13.8 to −13.6‰, whereas HO and SOB both have approximately 2‰ less negative δ^13^C values of around −11.5‰ (Fig. 2, Table 1). These between-site differences in δ^13^C are significant (Kruskal-Wallis test: H (3, N = 22) = 14.395; *p* = 0.002).

**Table 1:** Mean enamel δ^13^C and δ^18^O values of *H. primigenium* teeth.

Specimen no.	Tooth	Locality	n	δ^18^O_c_VPDB[‰]	σ	δ^13^C_c_VPDB[‰]	σ	CO_3_[wt. %]
PW 2000/10153	M2	EP	9	−9.0	0.20	−14.2	0.14	5.6
PW 2000/10171	M2	EP	14	−8.2	1.34	−14.6	0.30	6.1
PW 2000/10072	M2	EP	1	−8.7	0.1	−14.5	0.06	4.4
NMM (FZ EQ EP 1)	M	EP	1	−5.8	0.03	−12.4	0.04	5.1
NMM (FZ EQ EP 2)	M	EP	1	−6.6	0.04	−13.0	0.04	4.4
NMM (FZ EQ EP 3)	M	EP	1	−6.7	0.03	−13.7	0.02	5.4
NMM (FZ EQ EP 4)	M	EP	1	−7.2	0.08	−14.1	0.03	5.1
**Mean value EP**			**7**	−**7.5**	**1.1**	−**13.8**	**0.7**	**5.2**
HW164/55	P4	HO	7	−6.4	0.44	−11.0	0.11	4.7
HW153/55	P3	HO	1	−4.8	0.11	−11.1	0.10	4.7
HW755/59	M1	HO	7	−8.4	0.71	−12.2	0.19	5.4
HMLD Hip. III 53	M	HO	1	−5.9	0.09	−12.5	0.05	4.1
UMz P 1913	M	HO	1	−4.6	0.05	−12.7	0.06	4.4
SMNK Höw 06/117	M	HO	15	−6.0	0.42	−11.5	0.32	4.1
SMNK Höw 06/102	M	HO	1	−5.9	0.07	−11.5	0.07	4.4
SMNK Höw 06/55	M3	HO	15	−4.5	0.64	−10.2	0.29	4.8
**Mean value HO**			**8**	−**5.4**	**0.7**	−**11.5**	**0.8**	**4.6**
CM 600	P4	CH	15	−7.8	0.58	−14.5	0.44	5.3
CM 274	M3	CH	15	−8.4	0.62	−13.7	0.23	5.6
CM 574	M2	CH	19	−9.1	0.49	−13.4	0.16	5.8
CM 457	P3	CH	13	−7.3	0.84	−12.6	0.32	5.0
**Mean value CH**			4	−**8.2**	0.7	−**13.6**	0.7	**5.4**
FSL SOB−1	M3	SOB	1	−5.6	0.08	−11.2	0.10	6.4
FSL SOB−2	M3	SOB	1	−6.4	0.09	−11.7	0.07	6.1
FSL SOB−3	M3	SOB	1	−4.8	0.10	−11.4	0.08	5.1
**Mean value SOB**			**3**	−**5.6**	**0.7**	−**11.5**	**0.2**	**5.9**

**Figure 3:**
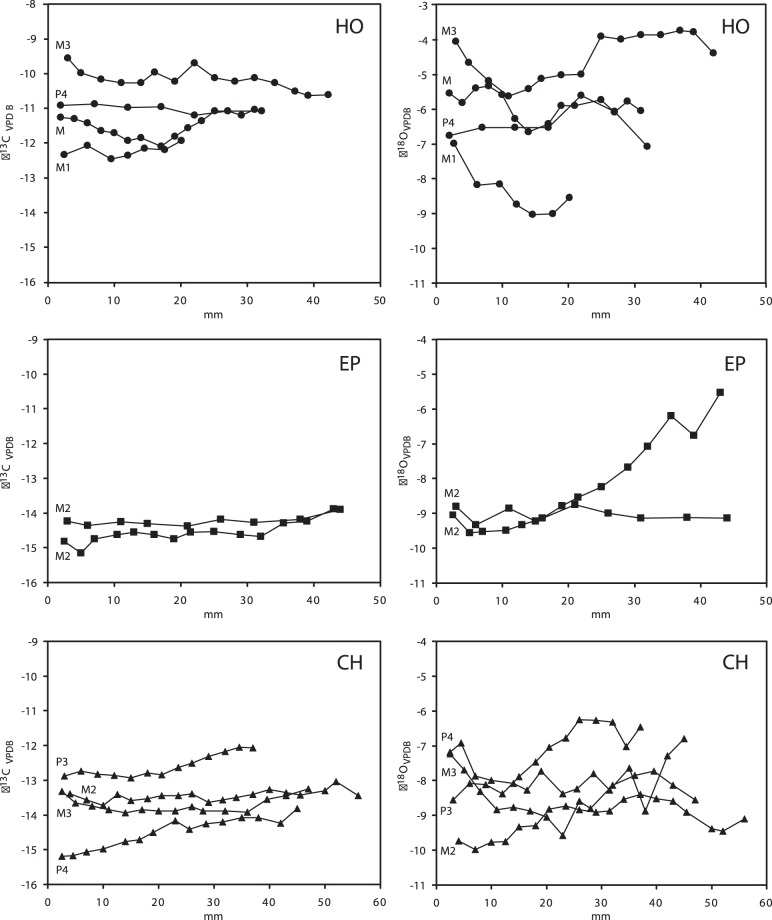
Intra-tooth variation of the enamel δ^13^C and δ^18^O values in 10 serially sampled teeth of *Hippotherium primigenium* molar and premolar teeth from Charmoille (CH), Eppelsheim (EP), and Höwenegg (HO). Samples are plotted against the distance from the crown in mm.

Intra-tooth δ^13^C variability of all 10 serially sampled premolars and molars from EP, CH and HO is nearly 6‰ (range: −15.2 to −9.5‰), but is small within a single tooth 0.9±0.3‰ (range: 0.5 to 1.4‰, Tables S1–S3). The teeth do not have clear seasonal trends in δ^13^C values (Fig. 3). Inter-tooth variability of mean enamel δ^13^C values in a single site is higher than the seasonal variation within a single tooth, with a range between 1.9 and 2.5‰, except for SOB which has a lower value of 0.5‰. The total range of δ^13^C values for all 22 teeth is 4.4‰. The δ^13^C values for the teeth of the HO and SOB populations are less negative than those of the other two *H. primigenium* populations from EP and CH (Fig. 2). Thus the HO and SOB hipparionines have ingested more ^13^C-enriched C_3_ plants compared to those of the latter localities.

The mean δ^18^O_CO3_ values of the enamel carbonate of the 22 *H. primigenium* teeth analyzed from the four localities range from –9.1 to –4.5‰ (Table 1). These between-population differences in δ^18^O_CO3_ are significant (Kruskal-Wallis test: H (3, N = 22) = 10.624; *p* = 0.013). The intra-tooth δ^18^O variation of all of the 10 serially sampled premolars and molars has a similar total range from –10.0 to –4.3‰ (Tables S1–S3). Intra-tooth variations of δ^18^O_CO3_ values are small and range from 0.6 to 2.3‰ with an average of 1.7±0.7‰. One tooth from EP (PW 10153) has a much higher variability οφ 4.0‰, which is likely a result of diagenetic alteration in the lower, less mineralized crown part of this unerupted M2 that also gave the highest δ^18^O_CO3_ values (Fig. 3). Some of the teeth show a seasonal trend in δ^18^O values but most intra-tooth δ^18^O patterns are relatively flat (Fig. 3). The HO and SOB hipparionines, which have the highest enamel δ^13^C values, also have the highest δ^18^O_CO3_ values (Fig. 2), hence they ingested water more enriched in ^18^O than that of the other localities.

The seven molars analyzed from Eppelsheim have the lowest mean δ^13^C values of –14.6 to –12.4‰ and the intra-tooth δ^13^C variation of the two serially sampled M2 have a range from 0.5 to 1.3‰. Mean δ^18^O_CO3_ values range from –9.0 to –5.8‰ while the intra-tooth variation of the oxygen isotope composition is the largest of all four investigated localities ranging from 0.5 to 3.2‰ (Table 1, Fig. 3).

The eight teeth from Höwenegg (P4, P3, M1 and M3 and four other molars) have the highest δ^13^C values of all teeth analyzed from all the localities, with a range from –12.7 to –10.2‰ (Fig. 2), while the intra-tooth variation of the four serially sampled teeth is low, from 0.3 to 1.1‰. The HO population has 1.5 to 2.5‰ less negative δ^13^C values compared to the EP and CH populations but values similar to SOB. δ^18^O_CO3_ values for all teeth range from –6.4‰ to –4.5‰ while the intra-tooth variation is in the range of 1.3 to 2‰ (Table 1, Fig. 3).

The four serially sampled teeth from Charmoille (P3, P4, M2 and M3) have low mean δ^13^C values of –14.6 to –12.6‰ similar to those of EP and the intra-tooth variation of the δ^13^C values ranges from 0.6 to 1.4‰. Mean δ^18^O_CO3_ values for all teeth has a range from –9.1 to –7.3‰ and the intra-tooth variation is 1.6 to 2.3‰ (Table 1, Fig. 3).

For Soblay only three molars were analyzed in bulk but no serial intra-tooth sampling was performed. δ^13^C values have only a narrow range from –11.7 to –11.2‰ and δ^18^O_CO3_ values range from –6.4 to –4.8‰. Teeth from SOB have less negative δ^13^C and δ^18^O_CO3_ values than the two EP and CH populations, similar to those of HO teeth.

### Dental Micro- and Mesowear Signatures

For microwear analysis, specimens of *Hippotherium primigenium* from the four investigated localities were integrated into a model constructed using present day grazing and browsing ungulates through a discriminant analysis (DA). An overall misclassification for extant species is about 11.54% (5.88% for browsers and 20.49% for grazers; Table S4). As expected, the sample of present-day browsers significantly differs from grazers in DA coordinates (*t*-test, *p*<0.001). Table S4 provides the coefficients of linear discriminant plus the correlation between discriminating variables and discriminant function.

**Figure 4:**
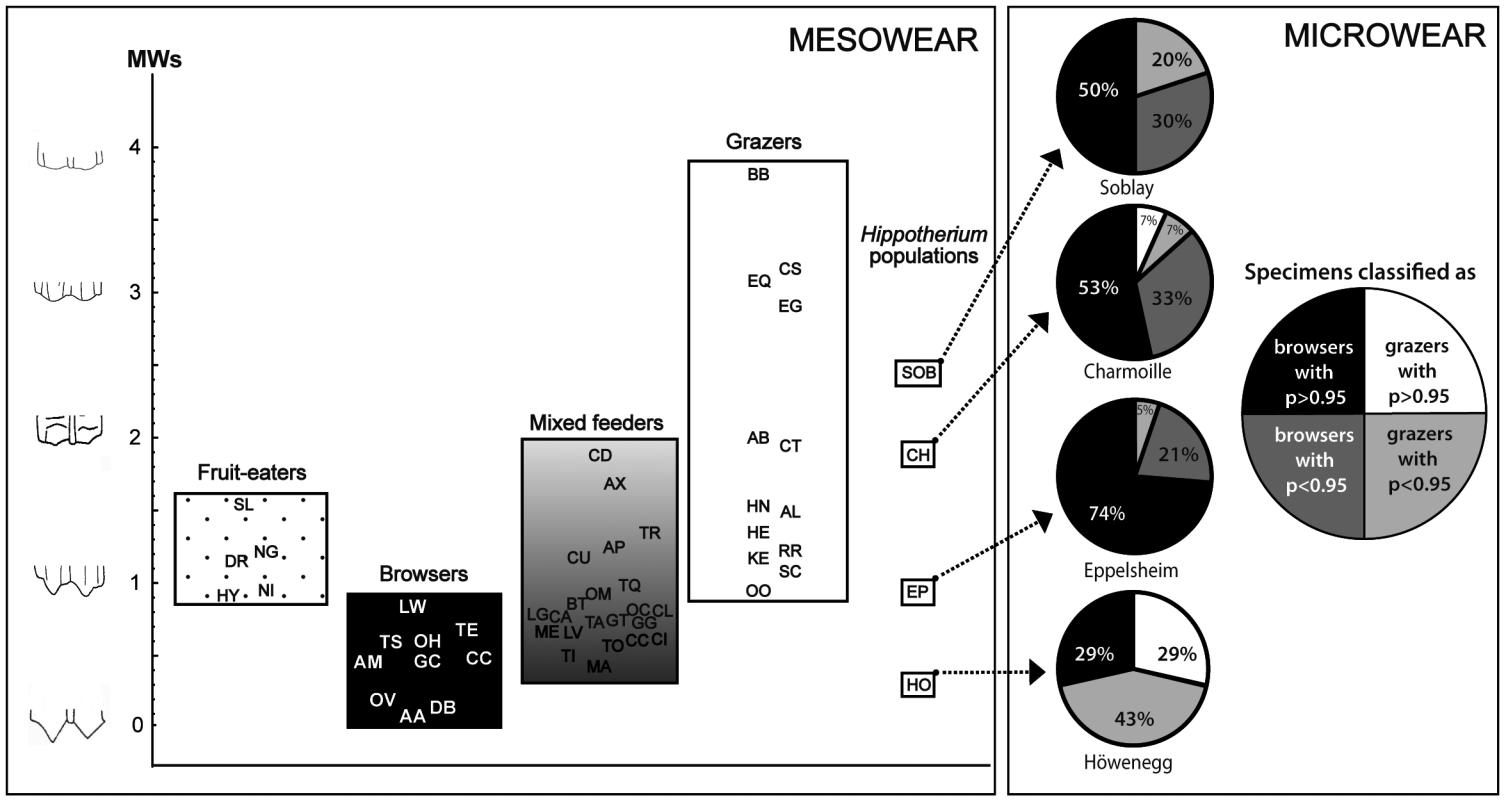
Molar mesowear and microwear patterns of the four populations of *Hippotherium primigenium*. Comparative extant species for microwear analysis are shown in Table S4 and in Table S5 for mesowear scoring.

Table 2 and Figure 4 display the classification of extinct hipparionines within this model. Hipparionines from the four Late Miocene localities investigated were not grazers but rather had mixed feeding habits with a strong dominance of browsing. Only one specimen out of 31 from the EP population has a dental microwear pattern similar to extant grazers, most of the remainder having similarities with living browsers. CH and SOB molar microwear patterns also share browsing characteristics. This contrasts with the HO sample for which 5 out of 7 individuals are classified as grazers, whereas the other two fit clearly with extant browsers. The differences in the distribution between the four populations along the discriminant analysis are valid (Kruskal-Wallis test: H (3, N = 51) = 9.975; *p* = 0.018).

**Table 2:** Number of individuals n mean m and standard deviation *sd* of the three main dental micro-wear variables (Number of scratches Ns, pits Np and percentage of pits Pp) for the four investigated populations (and sub-groups defined through the discriminant analysis) of *Hippotherium primigenium.*

				Ns		Np		Pp	
				m	sd	m	sd	m	sd
**Charmoille**	**Complete population**		**15**	**22.4**	**10.1**	**41.9**	**14.5**	**64.9%**	**12.5%**
	*Specimens classified in sub-groups:* *	*B95*	*8*	*18.0*	*6.5*	*48.0*	*16.8*	*71.9%*	*10.0%*
		*B50*	*5*	*21.8*	*5.4*	*35.8*	*9.1*	*61.9%*	*6.9%*
		*G50*	*1*	*49.0*	–	*33.0*	–	*40.2%*	–
		*G95*	*1*	*34.0*	–	*33.0*	–	*49.3%*	–
**Eppelsheim**	**Complete population**		**19**	**14.8**	**7.8**	**53.6**	**17.9**	**76.4%**	**15.5%**
	*Specimens classified in sub-groups:*	*B95*	*14*	*13.7*	*4.4*	*59.1*	*12.2*	*80.6%*	*7.6%*
		*B50*	*4*	*12.5*	*7.7*	*41.8*	*24.9*	*71.5%*	*22.8%*
		*G50*	*1*	*40*	–	*23*	–	*36.5%*	–
		*G95*	*0*	–	–	–	–	–	–
**Höwenegg**	**Complete population**		**7**	**29.1**	**9.2**	**31.7**	**28.8**	**44.5%**	**26.8%**
	*Specimens classified in sub-groups:*	*B95*	*2*	*20.0*	*12.7*	*70.0*	*21.2*	*77.0%*	*16.3%*
		*B50*	*0*	–	–	–	–	–	–
		*G50*	*3*	*30.7*	*6.1*	*23.3*	*4.7*	*43.2%*	*0.6%*
		*G95*	*2*	*36.0*	*2.8*	*6.0*	*4.2*	*14.1%*	*9.6%*
**Soblay**	**Complete population**		**10**	**28.1**	**8.3**	**50.8**	**17.7**	**63.0%**	**12.4%**
	*Specimens classified in sub-groups:*	*B95*	*5*	*23.0*	*6.8*	*64.8*	*8.2*	*74.0%*	*5.1%*
		*B50*	*3*	*35.3*	*7.8*	*43.0*	*12.3*	*54.6%*	*1.6%*
		*G50*	*2*	*30.0*	*4.2*	*27.5*	*3.5*	*47.8%*	*0.3%*
		*G95*	*0*	–	–	–	–	–	–

* Each (browsing and grazing) diet category is divided in two sub categories depending on the probability associated with the classification. Specimens that plot with either browsers (with p>0.95; B95) or grazers (with p>0.95; G95) browse and graze, respectively, the few weeks preceding the death. Two other subcategories are defined as browsers with 0.50>p>0.95 (B50) and as grazers with 0.50>p>0.95 (G50).

Mesowear data indicate a wide range of values from high attrition-dominated mesowear signatures (score = 0.22) to high-abrasion dominated mesowear scores (score = 2.11; Table 3 and Fig. 4). The most attrition-dominated wear signature is found in the HO population of *H. primigenium*, which plots in the central portion of the browser spectrum. In contrast, the SOB sample has the highest abrasion-dominated signature and therefore plots within the range of extant grazing species. The CH sample plots close to the SOB sample with a molar mesowear score of 1.94. The EP population plots within the mixed feeder range sandwiched between the spectrum for browsers and grazers (Fig. 4). Note that the EP score also falls within the range of fruit-eaters.

**Table 3:** Frequency of hipparionine specimens in the different mesowear categories (in percentage) and mesowear scores.

Populations	HS*	HR	LS	LR	LB	score
Höwenegg	80.00	20.00	0.00	0.00	0.00	0.22
Eppelsheim	18.75	79.17	0.00	2.08	0.00	0.86
Charmoille	9.09	39.39	6.06	39.39	6.06	1.94
Soblay	0.00	47.06	0.00	47.06	5.88	2.11

* HS = Combination of high relief H and sharp cusp S (score = 0), HR = combination of high relief H and round cusp R (score = 1), LS = combination of low relief L and sharp cusp S (score = 2), LR = combination of low relief L and round cusp R (score = 3), LB = combination of low relief L and blunt cusp B (score = 4). Average score per population is computed by the mean of the average score per individual in case that several teeth represent an individual.

## Discussion

### Feeding Ecology of the Earliest European Hipparionines

All *H. primigenium* teeth of Charmoille, Soblay, Eppelsheim and Höwenegg have low enamel δ^13^C values in the range expected for C_3_ feeders. Thus C_4_ plants were not a significant part of the diet of *Hippotherium*, confirming that C_4_ grasses were absent in Europe during the Late Miocene [96], [97]. Whether *H. primigenium* were C_3_ browsers or C_3_ grazers can not be inferred solely from their carbon isotopes. The small intra-tooth variability of enamel δ^13^C values of ≤1.4‰ indicates only small seasonal δ^13^C variations within the C_3_ plants ingested by *Hippotherium*. Intra-site differences of mean enamel δ^13^C values are somewhat larger (2 to 2.5‰). These differences in enamel δ^13^C values of the investigated *H. primigenium* teeth thus reflect δ^13^C variations of the dietary C_3_ plants (either C_3_ dicots or C_3_ monocots) due to seasonal and/or habitat differences. The enamel δ^13^C values of 60% of the *H. primigenium* teeth are 1 to 3‰ lower than the mean value of around –11.5‰ expected for herbivores feeding on C_3_ plants during the Miocene [98].

Low enamel δ^13^C values ≤ –13‰ for many of the *H. primigenium* teeth, especially those from EP and CH, suggest feeding was predominantly in forested ecosystems. This is compatible with the palaeoenvironmental reconstructions for these settings based on palaeofloral remains (see Text S1 for details). Forest-dwelling browsers are, due to canopy related effects, expected to have lower δ^13^C values compared to more open country or grassland browsers or grazers [55], [99]. However, *H. primigenium* was clearly not feeding in a dense evergreen tropical forest with a very strong canopy effect, otherwise much more negative δ^13^C enamel values would be expected [55].

In contrast, *H. primigenium* teeth from the HO and SOB populations have around 2‰ higher δ^13^C enamel values when compared to those of EP and CH (Fig. 2). This is probably not related to diagenetic alteration as enamel and dentin of the same teeth for the HO sample have distinct differences in their δ^13^C values of about 5‰ (Fig. S1). Thus the dentin has higher δ^13^C values, that is, values similar to those of the embedding sediment while the enamel has likely preserved original values. Therefore, the higher enamel δ^13^C values of the HO and SOB specimens indicate the ingestion of more ^13^C enriched C_3_ plants. This may reflect palaeoenvironmental effects (e.g., water stress, habitat openness) on the plants eaten. An alternative interpretation would be that the HO and SOB *H. primigenium* populations fed predominantly on different plants or plant parts (e.g., more woody plant remains or fruits) with less negative δ^13^C values. The HO and SOB specimens also have higher enamel δ^18^O values (Fig. 2) compared to the teeth of EP and CH, supporting the suggestion of environmental effects on the ingested plants, and possibly more intense water stress in the HO and SOB palaeoenvironments than in the vicinities of EP and CH. However, for SOB the palaeontological evidence indicates a humid and forested environment in the vicinity [23] and, therefore, does not support water stress. Interestingly, the dental mesowear patterns of the SOB population are consistent with the dental wear pattern of extant grazers (Fig. 4), which would not be expected for a forest dweller. Conversely, more than half of the Soblay specimens (7 out of 10) had molar microwear patterns similar to the browsing species (Table 2 and Figure 4). Thus the SOB *H. primigenium* grazed and browsed. This suggests that more open and drier areas with grass existed in the vicinity of the humid forested environment and that both settings were likely used for foraging by the SOB population. Predominant grazing best explains the higher δ^13^C and δ^18^O values and dental mesowear signatures of the SOB teeth. Feeding on canopy-derived fruits and leaves that have higher δ^13^C values than subcanopy plants could be an alternative explanation for higher enamel δ^13^C values in frugivorous forest dwelling taxa compared to subcanopy browsers [55]. As fruits and seeds tend also to be enriched in ^18^O relative to the source water [100], frugivores have higher enamel δ^18^O values. However, the abrasion dominated mesowear patterns of SOB do not comply with extensive frugivory (Fig. 4).

The HO population has similar δ^13^C and δ^18^O values compared to those of the SOB (Fig. 2). In contrast to the SOB population, the dental mesowear of the HO population resembles dietary reference taxa of present-day browsers (Fig. 4). However, molar microwear analysis detects some grazing habits for this population. This clearly points to a large dietary flexibility in *Hippotherium* on a meal-by-meal basis and thus its ability to succeed in different environments as a mixed feeder with opportunistic preferences for either browsing or grazing, depending on resource availability.

The low mean δ^13^C values of ≤ –13.6‰ for EP and CH (compared with SOB and HO) are compatible with browsing on C_3_ plants in a forested environment. The dental mesowear signature of the EP population supports mixed feeding habits, whereas, the dental mesowear analysis on the CH sample indicates a diet richer in grasses. However, note that such an abrasion-dominated mesowear signal could also be due to high fruit consumption. The hypothesis of grazing traits is challenged by the dental microwear results of these two populations. In fact, only one individual out of 19 from EP and two of 15 from CH can be considered as grazers; the remaining individuals all plot in the browsers group (Table 2 and Figure 4). These individuals obviously did not graze a considerable amount during the weeks or even months before their death.

Altogether the data support opportunistic and mixed feeding habits for *H. primigenium.* Indeed, long-term (molar mesowear patterns and bulk stable isotope compositions) and short-term (molar microwear patterns and serial intra-tooth stable isotope compositions) signals do not always corroborate each other and vary from one population to another and even within a single population. Given the different food properties and timescales represented by the various diet proxies this is not surprising. However, the data clearly demonstrate the potential of combined dental wear and stable isotope analysis to obtain refined and complementary dietary reconstructions of herbivores. This multi-proxy approach reveals dietary flexibility and enables us to gain new insights into the palaeoecology of extinct taxa and their feeding behaviour.

### The Habitat of the Earliest European Hipparionines

Differences in vegetation density and/or water stress may explain some of the dietary differences between the *H. primigenium* populations. For instance, the EP habitat was part of the Rhine River stream system in a flat and well-watered landscape. Water stress thus likely played a much less pronounced role at EP. Similarly, this may have been the case for HO as it was situated near a lake in the Hegau volcanic field on a tuff-dominated bedrock and well-drained limestone bedrock. Given the higher enamel δ^13^C and δ^18^O values from HO, and also SOB, compared to EP and CH, the HO and SOB populations may represent open landscape populations, which in turn would be expected to have had a more grass-dominated dietary regime. This is in agreement with the microwear results from HO teeth, but not for those from SOB where there is robust evidence for wet and forested habitat [23]. Note, however, that the SOB population has the most abrasion-dominated mesowear pattern of all four populations, probably indicating an overall grass-dominated diet which is in agreement with a more open habitat. In turn, the EP and CH habitats would be considered to have been more closed, and therefore less likely to provide access to grass and other abrasive food items. However, tooth mesowear patterns indicate that EP and CH hipparionines included abrasive components in their diet (grasses, grass-like plants). *Hippotherium primigenium* was thus generally a flexible and opportunistic meal-by-meal mixed feeder highly engaged in browsing, grazing and occasional frugivory and therefore was predominantly a forest dweller during the Late Miocene of Western Europe.

### General Implications for Palaeodiet and Palaeoecology of Hippotherium Primigenium

Despite the correlation of crown height with ecological factors relating to diet in modern herbivorous mammals, the correlation cannot prove that crown height is always a reliable predictor of diet in terms of browsing versus grazing [101]. Mihlbachler & Solounias [101] reported a less direct coevolution of diet and crown height in the fossil record of North American Merycoidodontidae compared to previous studies. The question of evolutionary trajectories which may also be responsible for an increase in crown height has been raised a number of times over the last decade, and a reappraisal of the question may now be required. One could be derived from the current data regarding the broad dietary flexibility of *H. primigenium.*

Because of their hypsodonty, ‘*Hipparions*’ were widely considered as grazers. However, there is another biological benefit of hypsodonty beyond the adaptation to an abrasive (grass) diet; a greater dietary flexibility. The present data indicate that *Hippotherium* had a variable C_3_ plant-based diet ranging from browsing to grazing and was thus a flexible and opportunistic mixed-feeder. At around 11 Ma, when hypsodont hipparions arrived in Eurasia, C_4_ plants, which comprise the majority of abrasive grasses in extant open environments, were absent or just a minor component in the global flora and did not become widespread until 8 to 6 Ma [19], [20]. This indicates that hipparions must have inhabited a Europe with an absence of C_4_ grasslands during the early Late Miocene (MN9) and could only have met their dietary needs with the C_3_ environment, which was probably forested. *Hippotherium* would have predominantly fed on available leafy plants such as trees and shrubs within these forested landscapes of Europe but could also switch to a more abrasive grass or even a fruit-based diet. This ecologic flexibility enabled hypsodont *Hippotherium* to feed both on abrasive and less abrasive food items, depending on their availability. This was advantageous for survival in environments with changing seasonality as would have occurred following the Mid-Vallesian crisis, with drier and more seasonal climates prevailing in Europe in the Late Miocene [102], [103].

Furthermore, opportunistic mixed-feeding may have facilitated the dispersal of *H. primigenium* via Asia due to any potential climatic (arid areas) and geographic barriers being easier to cope with due to its flexible diet. The North American ancestors of the earliest Eurasian hipparions from the *Cormohipparion* clade are best known from the Early Clarendonian (ca 12 Ma) quarries in Texas and Nebraska [2]. However, the population from which the first Eurasian individuals derived must have existed in Alaska, which would have been a relatively cool and humid place compared to the North American interior [104]. Conversely, preliminary results indicate that habitat conditions were quite dry as far south as central China in the earlier part of the Late Miocene [105]. Fortelius et al. [104] postulate a westward migratory route of earliest Old World hipparions, and consider “traditional” migratory dispersal routes unlikely because of aridity. However, the dietary signatures of this study of the first three-toed horses in Europe are indicative of a variety of dietary sources. Indeed, *Hippotherium primigenium* was likely able to graze as well browse, much in the same way as present-day wild asses. This dietary flexibility would explain its rapid dispersal into Eurasia despite climatic and environmental dispersal barriers, and thus was not necessarily linked to the expansion of grasslands for grazing. The hypsodonty of *H. primigenium* can thus be considered an example of Liem’s paradox in which a specialized phenotype (high crowned cheek teeth) enabled a more flexible mixed-feeding diet.

Given how wide the dietary niche of *H. primigenium* appears to be, its hypsodont dentition does not restrict it to a habitat of abrasive diets. This is also compatible with the reasoning that fallback foods may have more selective force in natural selection than preferred foods [106]. The hypsodont adaptation would allow *H. primigenium* to survive in a complex variety of landscapes, which did not necessarily provide the food items that limit hypsodont herbivores to abrasive diets. *Hippotherium* is thus another example that hypsodonty, although originally an adaptation to abrasive food, can enable a species to become a generalist feeder of both high- and low-abrasion diets, similarly to camelids [107], rhinocerotids [108], and other equids [12]. Finally, hypsodonty was advantageous for *H. primigenium* to expand its habitats and food resources in the increasingly drier and more open Late Miocene landscapes of Europe [109].

The appearance of *Hippotherium* in Europe as a hypsodont, opportunistic mixed feeder put competitive pressure on the brachydont equid *Anchitherium aurelianense* which was feeding in the same ecological niche [110]. This may have contributed to the decline and extinction of *Anchitherium* in the Old World following the Mid-Vallesian crisis, with only a short period of coexistence in the upper Vallesian (MN9) [111], [112]. The number of fossil sites with co-occuring *H. primigenium* and *A. aurelianense* specimens are thus limited, hampering a direct diet comparison of sympatric individuals. A rapid replacement of *Anchitherium* by *Hippotherium* seems quite plausible due to the latter having a high ecological tolerance; because of its hypsodonty it was capable of feeding on abrasive diets. Although some *Anchitherium* populations in arid areas such as Spain also have an adaptive increase in crown height, those in humid central European areas (i.e. Germany) did not, and they remained brachydont [113]. Overall, the hypsodont *H. primigenium* was able to live and feed in a variety of habitats and therefore was a very successful migrant species.

## Conclusions

To better understand the Late Miocene dispersal of the earliest high-crowned hipparionines in the Old World two hypotheses were investigated: (1) hypsodont cheek teeth facilitated the exploitation of grassy abrasive vegetation in evolving open grassland landscapes and, (2) hypsodont cheek teeth enabled *H. primigenium* to occupy a broad dietary niche. Combined dental meso- and microwear together with enamel oxygen and carbon stable isotope analyses on four populations of hipparionines in Europe, amongst the earliest in the Old World, support mixed feeding habits for *H. primigenium* with a strong reliance on leafy plants rather than on grazing habits. This high-crowned equid was thus adapted to use a wide range of dietary sources and had a large dietary flexibility. Feeding changed partly on a meal-by-meal basis, but was likely also affected by local and seasonal availability of vegetational resources in the natural food supply. This dietary flexibility was likely a key factor for the rapid dispersal of *H. primigenium* from North America into Eurasia, given the different environmental settings in this region.

With the broad feeding habits of *H. primigenium*, its hypsodont dentition may no longer be regarded as a constraint for forage on abrasive items in open landscapes. Instead, it would rather enable opportunistic feeding from grazing to pure browsing for long periods of time; even when no less abrasive diets were available. The hypsodonty of its cheek teeth would allow *H. primigenium* to survive in a variety of landscape types which did not necessarily provide the food items that would limit hypsodont herbivores to abrasive diets. Thus *Hippotherium* is an example of the Liem’s paradox, hypothesizing that specialized phenotypes (here hypsodonty) enable more ecological flexibility in a taxon and the occupation of a broader dietary niche.

The arrival of this ecologically flexible equid must have imposed severe competition on brachydont anchitheres that occupied a variable browsing or mixed feeding niche in the Late Miocene of Europe [110]. The higher permanent dietary flexibility of *H. primigenium* might thus be the cause for the rapid replacement of the low-crowned equid *Anchitherium aurelianense* in Europe during the Late Miocene (MN9–MN10).

## Materials and Methods

We thank the Forschungsinstitut und Naturmuseum Senckenberg (Frankfurt), the Staatliches Museum für Naturkunde (Karlsruhe), and the Landesmuseum Rheinland-Pfalz (Mainz), the Collections d’Histoire Naturelle de l’Université de Lyon (A. Prieur) and the Naturhistorisches Museum (Basel, L. Costeur) for access to the fossil specimens investigated in this study. All the specimens were loaned in the aforementioned museum collections and returned after analysis.

### Carbon and Oxygen Isotope Analysis of Tooth Enamel

Serial and bulk enamel samples of isolated cheek teeth of *Hippotherium primigenium* from Eppelsheim (EP; n = 7), Höwenegg (HO; n = 8), and Charmoille (CH; n = 4) were analysed for their carbon and oxygen isotopic composition of carbonate in the apatite (Table 1). If possible, only teeth such as P4, M2 and M3, which formed after weaning, were analyzed. Bulk enamel samples of isolated cheek teeth of *Hippotherium primigenium* from Soblay (SOB; n = 3) were also analysed for their carbon and oxygen isotope composition of carbonate in the apatite (see Table 1 for details). For isotope analyses teeth with the least possible wear were selected to sample the complete tooth length and thus longest growth period possible. Due to limited sample availability, teeth of different jaw positions were sampled for the isotopic measurements (Table 1). The tooth cement was removed and the enamel surface mechanically cleaned prior to enamel sampling. Bulk enamel samples were recovered by drilling a line parallel to the growth axis of the tooth along the entire length from the crown to the root. Serial enamel samples were hand-drilled with a Proxxon drill perpendicular to the growth axis on a non-occlusal tooth surface. Six teeth, two per locality for HO, EP, and CH, were serially sampled in 3-mm intervals perpendicular to the growth axis to investigate the seasonal intra-tooth variation of the carbon and oxygen isotopic composition (Table S1–S3).

The enamel powder was pretreated according to Koch et al. [114]. 10 mg enamel powder were soaked for 24 hours with 2% NaOCl and 1 M calcium-acetate buffer solution for 24 hours in a powder/solution ratio of 0.04 g/ml to remove organic substances and diagenetic carbonates, respectively, prior to analysis of the carbon (δ^13^C) and oxygen (δ^18^O_CO3_) isotopic composition of the carbonate in the apatite. About 2 mg pretreated enamel powder was reacted with 100% H_3_PO_4_ for 90 minutes at 70°C using a ThermoFinnigan Gasbench II [115]. Carbon and oxygen isotope ratios of the generated CO_2_ were measured in continuous flow mode on a Finnigan Delta Plus XL isotope ratio gas mass spectrometer at the University of Lausanne and Tübingen. For this reaction the same acid fractionation factor as between calcite and CO_2_, was assumed to be applicable. The measured carbon and oxygen isotopic compositions were normalized to the in-house Carrara marble calcite standard that has been calibrated against the international NBS-19 calcite standard. The isotope composition of tooth enamel apatite is reported in the usual δ-notation in per mil (‰) relative to the known isotope reference standard VPDB with δ^13^C or δ^18^O (‰) = [(R_sample_/R_standard_) −1]×1000, where R_sample_ and R_standard_ are the ^13^C/^12^C and ^18^O/^16^O ratios in the sample and standard, respectively. Precision for the carbon (δ^13^C) and oxygen (δ^18^O) isotopic composition of carbonate in the apatite is better than ±0.1‰ and ±0.15‰, respectively. The NBS 120c Florida phosphate rock standard, also pre-treated after Koch et al. [114], gave values of δ^13^C_VPDB_ = –6.23±0.09‰ and δ^18^O_VPDB_ = –2.23±0.10‰ (n = 9).

### Microwear Analysis

After initial examination, many specimens were excluded from analysis because they were physically altered during transportation or compaction of sediments [116]. Since microwear patterns may vary from mesial to distal teeth, the analysis is preferentially restricted to upper and lower M1s and M2s. The dental microwear patterns of fossil taxa were compared to those of extant grazing and browsing ungulates including artiodactyls and perissodactyls (Tables S4 and Figure 4). All microwear signatures of this study were only determined by one experienced observer (G. Merceron) to avoid any potential inter-observer error [117].

Regarding dental microwear analysis, several protocols were developed to quantify occlusal wear patterns at a microscopic scale. Differences concern casting procedures applied and the data acquisition. In this study the protocol of Merceron et al. [118] is employed because it combines the high quality of light stereomicroscopy with reliable analyses of high-resolution digitized images. To assess the feeding preferences the hipparionines were compared with extant species using discriminant analysis (DA) (Tables S4 and Figure 4). This multivariate statistical technique first evaluates the ability of the microwear variables to discriminate grazing species from the browsers. The mere pure grazing and browsing present-day ungulates are here considered as model for the DA; no intermediate feeders are considered. The dental microwear pattern of individuals engaged in both grazing and browsing is similar either to that of browsers or to that of grazers depending on their last few meals. A set of 9 variables (Ns, Np, Ls, Pp, Tot, Nws, Nfs, Nlp, Nsp) was considered for running the DA [92]. Because the normality and homogeneity of variance are not guaranteed, the variables were log-transformed. DA requires the very same assumptions as the analysis of variance. Then, the extinct hipparionine specimens are classified into the browsing or the grazing kernel according to the model set up with living species (Figure 2 and Table S4). As suggested by DeGusta and Vrba [119], a threshold probability here set up at 5% is used to distinguish the significant predictions from more questionable reliability.

### Mesowear Analysis

Dental facet development on the molar surfaces of herbivorous ungulates appears to be strongly tied to feeding styles [86], [120]. Dental mesowear reflects the degree of attritive and abrasive wear on the molar and premolar occlusal surface. Attritive wear is due to the tooth/tooth contact and results in high crown relief and sharp cusp apices. Abrasive wear is due to tooth/food contacts, obliterates dental facets and results in lower crown relief and more round or blunted cusp apices. Initially dental mesowear analysis was based only on the second upper molars [86], however, Kaiser and Solounias [121] extended the analytical model to include three more maxillary tooth positions (P4, M1, and M3) in hypsodont equids. The mesowear method treats ungulate tooth mesowear as two variables: occlusal relief and cusp shape. Occlusal relief (OR) is classified as high (H) or low (L), depending on how high the cusps rise above the valley between them. The second mesowear variable, cusp shape, includes 3 scored attributes: sharp (S), round (R) and blunt (B) according to the degree of facet development. In addition to established mesowear convention, a combined mesowear score was computed from each population similar [110], [122]. The convention used, however differs from that used by Semprebon and Rivals [123] by inclusion of a score for the combination of low reliefs and sharp cusps. This combination is frequently found especially in grazers, but was not accommodated by Semprebon and Rivals [123]. A combination of high relief and sharp cusps was assigned a score of “0”, a combination of high relief and round cusp was assigned a score of “1”, a combination of low relief and sharp cusp was assigned a score of “2”, a combination of low relief and round cusp was assigned a score of “3” and a combination of low relief and blunt cusps was assigned a score of “4”. In this convention, a score of 0 represents the most attrition-dominated mesowear signature, while a score of 4 would represent the most abrasion-dominated signature. Individual scores were averaged and a mean score was calculated for each species (Fig. 3). Scores thus indicate the over all abrasiveness of the diet a species has to cope with. As comparative dataset, we consider the medium-size ungulates (artiodactyls and perissodactyls) with sample size clustered in broad diet categories (Figure 4, Table S5).

## Supporting Information

Figure S1 **δ^13^C and δ^18^O values of enamel and dentin H. primigenium teeth from the Höwenegg locality as well as on sediment sample from the main fossil-bearing layer.** Note the large difference in δ^13^C values indicating a diagenetic alteration of the dentine while enamel still has values typical for C_3_ feeders.
(TIF)

Table S1 **C and O isotopic compositions of the serial sampled Eppelsheim H. primigenium teeth.**
(XLS)

Table S2 **C and O isotopic compositions of the Höwenegg H. primigenium teeth.**
(XLS)

Table S3 **C and O isotopic compositions of the Charmoille H. primigenium teeth.**
(XLS)

Table S4 **Summary statistics of the main dental microwear variables for the browsing and grazing ungulates that composed the comparative dataset.**
(DOC)

Table S5 **List of extant species used for mesowear analysis.**
(DOC)

Text S1 **Detailed site description of the fossil localites.**
(DOC)

## References

[1] Simpson GG (1951) Horses. Oxford: Oxford University Press. 323 p.

[2] MacFadden BJ (1992) Fossil Horses: Systematics, Paleobiology, and Evolution of the Family Equidae. Cambridge: Cambridge University Press. 369 p.

[3] MacFadden BJ (2005) Fossil horses - evidence for evolution Science. 307: 1728–1730. 10.1126/science.1105458 15774746

[4] Janis CM (2008) An evolutionary history of browsing and grazing ungulates. Ecological Studies 195: 21–45.

[5] Merceron G, Blondel C, Brunet M, Sen S, Solounias N, et al (2004) The late Miocene paleoenvironment of Afghanistan as inferred from dental microwear in artiodactyls. Palaeogeography, Palaeoclimatology, Palaeoecology 207: 143–163.

[6] Merceron G, Schulz E, Kordos L, Kaiser TM (2007) Paleoenvironment of *Dryopithecus brancoi* at Rudabánya, Hungary: evidence from dental meso- and micro-wear analyses of large vegetarian mammals. Journal of Human Evolution 53: 331–349. 17719619 10.1016/j.jhevol.2007.04.008

[7] Schulz E, Piotrowski V, Clauss M, Mau M, Merceron G, et al (2013) Dietary abrasiveness determines variability in microwear and dental surface texture in rabbits. PLoS One 8: e56167. 23405263 10.1371/journal.pone.0056167 PMC3566079

[8] Lucas PW, Omar R, Al-Fadhalah K, Almusallam AS, Henry AG, et al.. (2013) Mechanisms and causes of wear in tooth enamel: implications for hominin diets. Journal of Royal Society Interface 10. 10.1098/rsif.2012.0923 PMC3565742 23303220

[9] Sanson GD, Kerr SA, Gross KA (2007) Do silica phytoliths really wear mammalian teeth? Journal of Archaeological Science 34: 526–531.

[10] Hummel J, Findeisen E, Südekum K-H, Ruf I, Kaiser TM, et al (2011) Another one bites the dust: faecal silica levels in large herbivores correlate with hypsodonty. Proceedings of the Royal Society of London B 278: 1742–1747. 10.1098/rspb.2010.1939 PMC3081769 21068036

[11] Damuth J, Janis C (2011) On the relationship between hypsodonty and feeding ecology in ungulate mammals, and its utility in palaeoecology. Biological Reviews 86: 733–758. 21418504 10.1111/j.1469-185X.2011.00176.x

[12] Mihlbachler MC, Rivals F, Solounias N, Semprebon GM (2011) Dietary change and evolution of horses in North America. Science 331: 1178–1181. 21385712 10.1126/science.1196166

[13] Strömberg CAE (2002) The origin and spread of grass dominated ecosystems in the late Tertiary of North America: preliminary results concerning the evolution of hypsodonty. Palaeogeography, Palaeoclimatology, Palaeoecology 177: 59–75.

[14] Strömberg CAE (2006) Evolution of hypsodonty in equids: testing a hypothesis of adaptation. Paleobiology 32: 236–258.

[15] MacFadden BJ, Solounias N, Cerling TE (1999) Ancient diets, ecology, and extinction of 5-million-year-old horses from Florida. Science 283: 824–827. 9933161 10.1126/science.283.5403.824

[16] Woodburne MO (2009) The early Vallesian vertebrates of Atzelsdorf (Late Miocene, Austria). 9. *Hippotherium* (Mammalia, Equidae). Annalen des Naturhistorischen Museums in Wien, Serie A 111: 585–604.

[17] Harzhauser M (2009) The early Vallesian vertebrates of Atzelsdorf (Late Miocene, Austria). 2. Geology. Annalen des Naturhistorischen Museums in Wien, Serie A 111: 479–488.

[18] Strömberg CAE, Werdelin L, Friis EM, Sarac G (2007) The spread of grass-dominated habitats in Turkey and surrounding areas during the Cenozoic: phytolith evidence. Palaeogeography, Palaeoclimatology, Palaeoecology 250: 18–49.

[19] Cerling TE, Wang Y, Quade J (1993) Expansion of C4 ecosystems as an indicator of global ecological change in the late Miocene. Nature 361: 344–345.

[20] Cerling TE, Harris JR, MacFadden BJ, Leakey MG, Quade J, et al (1997) Global vegetation change through the Miocene/Pliocene boundary. Nature 389: 153–158.

[21] Liem KF (1980) Adaptive significance of intraspecific and interspecific differences in the feeding repertoires of cichlid fishes. American Zoologist 20: 295–314.

[22] Robinson BW, Wilson DS (1998) Optimal foraging, specialization, and a solution to Liem’s paradox. American Naturalist 151: 223–235. 10.1086/286113 18811353

[23] Ménouret B, Mein P (2008) Les vertébrés du Miocène supérieur de Soblay (Ain, France). Documents du Laboratoire de Géologie de Lyon 165: 1–97.

[24] Bernor RL, Koufos GD, Woodburne MO, Fortelius M (1996) The evolutionary history and biochronology of European and Southwest Asian Late Miocene and Pliocene Hipparionine Horses. In: Bernor RL, Fahlbusch V, Mittmann H-W, editors. The Evolution of Western Eurasian Neogene Mammal Faunas. New York: Columbia University Press. pp. 307–338.

[25] Grimm MC (2005) Beiträge zur Lithostratigraphie des Paläogens und Neogens im Oberrheingebiet (Oberrheingraben, Mainzer Becken, Hanauer Becken). Geologisches Jahrbuch Hessen 132: 79–112.

[26] Franzen JL (2011) Eppelsheim Formation. In: Grimm KI, editor. Stratigraphie von Deutschland IX Tertiär, Teil 1: Oberrheingraben und benachbarte Tertiärgebiete. Hannover: Deutsche Gesellschaft für Geowissenschaften. pp. 184–187.

[27] Woodburne MO, Bernor RL, Swisher CCI (1996) An appraisal of the stratigraphic and phylogenetic bases for the “Hipparion Datum” in the Old World. In: Bernor RL, Fahlbusch V, Mittmann HW, editors. The Evolution of Western Eurasian Neogene Mammal Faunas. New York: Columbia University Press. pp. 124–136.

[28] Steininger FF (1999) Chronostratigraphy, Geochronology and Biochronology of the Miocene “European Land Mammal Mega-Zones (ELMMZ)” and the Miocene “Mammal-Zones (MN-Zones)”. In: Rössner GE, Heissig K, editors. Land Mammals of Europe. München: Verlag Friedrich Pfeil. pp. 9–24.

[29] Andrews PA, Bernor RL (1999) Vicariance Biogeography and Paleoecology of Eurasian Miocene hominoid primates. In: Agusti J, Rook L, Andrews P, editors. The Evolution of Neogene Terrestrial Ecosystems in Europe. Cambridge: Cambridge University Press, pp. 454–488.

[30] Böhme M, Aiglstorfer M, Uhl D, Kullmer O (2012) The Antiquity of the Rhine River: Stratigraphic Coverage of the Dinotheriensande (Eppelsheim Formation) of the Mainz Basin (Germany). PLoS ONE 7: e36817. 22615819 10.1371/journal.pone.0036817 PMC3353959

[31] Franzen JL, Fejfar O, Storch G, Wilde V, editors (2003) Eppelsheim 2000 - new discoveries at a classic locality. Rotterdam: Deinsea. 217–234 p.

[32] Franzen JL (2000) Auf dem Grunde des Urrheins – Ausgrabungen bei Eppelsheim. Natur und Museum 130: 169–180.

[33] Swisher CC (1996) New ^40^Ar/^39^Ar dates and their contribution toward a revised chronology for the late Miocene of Europe and West Asia. In: Bernor RL, Fahlbusch V, Mittmann H-W, editors. The evolution of western Eurasian Neogene mammal faunas. New York: Columbia University Press. pp. 64–77.

[34] Munk W, Bernor RL, Heizmann EPJ, Mittmann HW (2007) Excavations at the Late Miocene MN9 (10.3 Ma) locality of Höwenegg (Hegau), southwest Germany, 2004–2006. Carolinea 65: 5–13.

[35] Kälin D (1997) Litho- und Biostratigraphie der mittel- bis obermiozänen Bois de Raube-Formation (Nordwestschweiz). Eclogae Geologicae Helvetiae 90: 97–114.

[36] Becker D (2003) Paléoécologie et paléoclimats de la Molasse du Jura (Oligo-Miocène): apport des Rhinocerotoidea (Mammalia) et des minéraux argileux. GeoFocus 9: 327.

[37] Sharp ZD, Cerling TE (1998) Fossil isotope records of seasonal climate and ecology: Straight from the horse’s mouth. Geology 26: 219–222.

[38] Hoppe KA, Stover SM, Pascoe JR, Amundson R (2004) Tooth enamel biomineralization in extant horses: implications for isotopic microsampling. Palaeogeography Palaeoclimatology Palaeoecology 203: 299–311.

[39] Bryant JD, Froelich PN, Showers WJ, Genna BJ (1996) Biologic and climatic signals in the oxygen isotopic composition of Eocene-Oligocene equid enamel phosphate. Palaeogeography, Palaeoclimatology, Palaeoecology 126: 75–89.

[40] Balasse M (2002) Reconstructing dietary and environmental history from enamel isotopic analysis: time resolution of intra-tooth sequential sampling. International Journal of Osteoarchaeology 12: 155–165.

[41] Fricke HC, O’Neil JR (1996) Inter- and intra-tooth variation in the oxygen isotope composition of mammalian tooth enamel phosphate: implications for palaeoclimatological and palaeobiological research. Palaeogeography, Palaeoclimatology, Palaeoecology 126: 91–99.

[42] Nelson SV (2007) Isotopic reconstructions of habitat change surrounding the extinction of *Sivapithecus*, a Miocene hominoid, in the Siwalik Group of Pakistan. Palaeogeography, Palaeoclimatology, Palaeoecology 243: 204–222.

[43] Tieszen LL, Fagre T (1993) Effect of diet quality and composition on the isotopic composition of respiratory CO_2_, bone collagen, bioapatite, and soft tissues. In: Lambert JB, Grupe G, editors. Prehistoric Human Bone: Archaeology at the Molecular Level. Berlin: Springer-Verlag. pp. 121–155.

[44] DeNiro MJ, Epstein S (1978) Influence of diet on the distribution of carbon isotopes in animals. Geochimica et Cosmochimica Acta 42: 495–506.

[45] Cerling TE, Harris JM, MacFadden BJ (1997) Carbon isotopes, diets of North American equids, and the evolution of North American C4 grasslands. In: Griffiths H, Robinson D, van Gardingen P, editors. Stable isotopes and the integration of biological, ecological, and geochemical processes. Oxford: Bios Scientific Publishers. pp. 363–379.

[46] Deines P (1980) The isotopic composition of reduced organic carbon. In: Fritz P, Fontes C, editors. Handbook of Environmental Geochemistry. New York: Elsevier. pp. 239–406.

[47] Cerling TE, Harris JM (1999) Carbon isotope fractionation between diet and bioapatite in ungulate mammals and implications for ecological and paleoecological studies Oecologia. 120: 347–363. 10.1007/s004420050868 28308012

[48] Passey BH, Robinson TF, Ayliffe LK, Cerling TE, Sponheimer M, et al (2005) Carbon isotope fractionation between diet, breath CO_2_, and bioapatite in different mammals. Journal of Archaeological Science 32: 1459–1470.

[49] Sullivan CH, Krueger HW (1981) Carbon isotope analysis of separate chemical phases in modern and fossil bone. Nature 301: 177–178. 10.1038/292333a0 7019719

[50] Lee-Thorp JA, van der Merwe NJ (1987) Carbon isotope analysis of fossil bone apatite. South African Journal of Science 83: 712–715.

[51] Quade J, Cerling TE, Barry JC, Morgan ME, Pilbeam DR, et al (1992) A 16-Ma record of paleodiet using carbon and oxygen isotopes in fossil teeth from Pakistan. Chemical Geology 94: 183–192.

[52] Mateu Andrés I (1993) A revised list of the European C4 plants. Photosynthetica 26: 323–331.

[53] Drucker D, Bocherens H, Bridault A, Billiou D (2003) Carbon and nitrogen isotopic composition of red deer (*Cervus elaphus*) collagen as a tool for tracking palaeoenvironmental change during the Late Glacial and Early Holocene in the northern Jura (France). Palaeogeography, Palaeoclimatology, Palaeoecology 195: 375–388.

[54] Feranec RS, MacFadden BJ (2006) Isotopic discrimination of resource partitioning among ungulates in C_3_-dominated communities from the Miocene of Florida and California. Paleobiology 32: 191–205.

[55] Cerling TE, Hart JA, Hart TB (2004) Stable isotope ecology in the Ituri Forest. Oecologia 138: 5–12. 14530961 10.1007/s00442-003-1375-4

[56] Tütken T, Vennemann T (2009) Stable isotope ecology of Miocene large mammals from Sandelzhausen, Germany. Paläontologische Zeitschrift 83: 207–226.

[57] Heaton THE (1999) Spatial, species, and temporal variations in the ^13^C/^12^C ratios of C_3_ plants: implications for palaeodiet studies. Journal of Archaeological Science 26: 637–649.

[58] Kohn MJ (2010) Carbon isotope compositions of terrestrial C3 plants as indicators of (paleo)ecology and (paleo)climate. Proceedings of the National Academy of Sciences of the United States of America 107: 19691–19695. 21041671 10.1073/pnas.1004933107 PMC2993332

[59] Diefendorf AF, Mueller KE, Wing SL, Koch PL, Freeman KH (2010) Global patterns in leaf ^13^C discrimination and implications for studies of past and future climate. Proceedings of the National Academy of Sciences of the United States of America 107: 5738–5743. 20231481 10.1073/pnas.0910513107 PMC2851872

[60] Longinelli A (1984) Oxygen isotopes in mammal bone phosphate: A new tool for palaeohydrological and palaeoclimatological research? Geochimica et Cosmochimica Acta 48: 385–390.

[61] Kohn MJ (1996) Predicting animal δ^18^O: Accounting for diet and physiological adaptation. Geochimica et Cosmochimica Acta 60: 4811–4829.

[62] Huertas AD, Iacumin P, Stenni B, Chillon BS, Longinelli A (1995) Oxygen isotope variations of phosphate in mammalian bone and tooth enamel. Geochimica et Cosmochimica Acta 59: 4299–4305.

[63] Dansgaard W (1964) Stable isotopes in precipitation. Tellus 16: 436–468.

[64] Rozanski K, Araguás-Araguás L, Gonfiantini R (1993) Isotopic patterns in modern global precipitaion. Geophysical Monograph 78: 1–36.

[65] Levin NE, Cerling TE, Passey BH, Harris JM, Ehleringer JR (2006) A stable isotope aridity index for terrestrial environments. Proceedings of the National Academy of Sciences of the United States of America 103: 11201–11205. 16840554 10.1073/pnas.0604719103 PMC1544065

[66] Tütken T, Vennemann TW, Janz H, Heizmann EPJ (2006) Palaeoenvironment and palaeoclimate of the Middle Miocene lake in the Steinheim basin, SW Germany: A reconstruction from C, O, and Sr isotopes of fossil remains. Palaeogeography, Palaeoclimatology, Palaeoecology 241: 457–491.

[67] Bryant JD, Froelich PN (1995) A model of oxygen isotope fractionation in body water of large mammals. Geochimica et Cosmochimica Acta 60: 4523–4537.

[68] Kohn MJ, Schoeninger MJ, Valley JW (1996) Herbivore tooth oxygen isotope compositions: Effects of diet and physiology. Geochimica et Cosmochimica Acta 60: 3889–3896.

[69] Ayliffe LK, Chivas AR (1990) Oxygen isotope composition of the bone phosphate of Australian kangaroos: Potential as a palaeoenvironmental recorder. Geochimica et Cosmochimica Acta 54: 2603–2609.

[70] Sponheimer M, Lee-Thorp JA (1999) Oxygen isotopes in enamel carbonate and their ecological significance. Journal of Archaeological Science 26: 723–728.

[71] Kohn MJ, Schoeninger MJ, Valley JW (1998) Variability in oxygen isotope compositions of herbivore teeth: reflections of seasonality or developmental physiology? Chemical Geology 152: 97–112.

[72] Nelson SV (2005) Paleoseasonality inferred from equid teeth and intra-tooth isotopic variability. Palaeogeography, Palaeoclimatology, Palaeoecology 222: 122–144.

[73] van Dam JA, Reichart GJ (2009) Oxygen and carbon isotope signatures in late Neogene horse teeth from Spain and application as temperature and seasonality proxies. Palaeogeography, Palaeoclimatology, Palaeoecology 274: 64–81.

[74] Balasse M, Ambrose SH, Smith AB, Price D (2002) The seasonal mobility model for prehistoric herders in the south-western Cape of South Africa assessed by isotopic analysis of sheep tooth enamel. Journal of Archaeological Science 29: 917–932.

[75] Gügel IL, Grupe G, Kunzelmann K-H (2001) Simulation of dental microwear: Characteristic traces by opal phytoliths give clues to ancient human dietary behavior. American Journal of Physical Anthropology 114: 124–138. 11169902 10.1002/1096-8644(200102)114:2<124::AID-AJPA1012>3.0.CO;2-S

[76] Teaford MF, Oyen OJ (1989) *In vivo* and *in vitro* turnover in dental microwear. American Journal of Physical Anthropology 80: 447–460. 2513725 10.1002/ajpa.1330800405

[77] Scott RS, Teaford MF, Ungar PS (2012) Dental microwear texture and anthropoid diets. American Journal of Physical Anthropology 147: 551–579. 22331579 10.1002/ajpa.22007

[78] Scott JR (2012) Dental microwear texture analysis of extant African Bovidae. Mammalia 76: 157–174.

[79] Merceron G, Escarguel G, Angibault J-M, Verheyden-Tixier H (2010) Can dental microwear textures record dietary inter-individual dietary variations? PLoS ONE 5(3): e9542. 20209051 10.1371/journal.pone.0009542 PMC2832010

[80] Ungar PS, Scott RS, Scott JR, Teaford MF (2008) Dental microwear analysis: historical perspectives and new approaches In: Irish JD, Nelson GC, editors. Volume on Dental Anthropology. Cambridge: Cambridge University. pp. 389–425.

[81] Ungar PS, Merceron G, Scott RS (2007) Dental microwear texture analysis of Varswater bovids and Early Pliocene paleoenvironments of Langebaanweg, Western Cape Province, South Africa. Journal of Mammalian Evolution 14: 163–181.

[82] Blondel C, Merceron G, Andossa L, Mackaye HT, Vignaud P, et al (2010) -(Chad) and early hominid habitats in Central Africa. Palaeogeography, Palaeoclimatology, Palaeoecology 292: 184–191.

[83] Rivals F, Mihlbachler MC, Solounias N (2007) Effect on the ontogenetic-age distribution in fossil and modern samples on the interpretation of ungulate paleodiets using the mesowear method. Journal of Vertebrate Paleontology 27: 763–767.

[84] Franz-Odendaal T, Solounias N (2004) Comparative dietary evaluations of an extinct giraffid (*Sivatherium hendeyi*) (Mammalia, Giraffidae, Sivatheriinae) from Langebaanweg, South Africa (Early Pliocene). Geodiversitas 26: 675–685.

[85] Kaiser TM (2003) The dietary regimes of two contemporaneous populations of *Hippotherium primigenuim* (Perissodactyla, Equidae) from the Vallesian (Upper Miocene) of Southern Germany. Palaeogeography, Palaeoclimatology, Palaeoecology 198: 381–402.

[86] Fortelius M, Solounias N (2000) Functional characterization of ungulate molars using the abrasion-attrition wear gradient: A new method for reconstructing paleodiets. American Museum Novitates 3301: 1–36.

[87] Solounias N, Teaford MF, Walker A (1988) Interpreting the diet of extinct ruminants: the case of a non-browsing giraffid. Paleobiology 14: 287–300.

[88] Kaufman PB, Dayanandan P, Franklin CI (1985) Structure and function of silica bodies in the epidermal system of grass bodies. Annals of Botany 55: 487–507.

[89] Lanning FC, Eleuterius LN (1989) Silica deposition in some C_3_ and C_4_ species of grasses, sedges, and composites in the USA. Annals of Botany 63: 395–410.

[90] Mac Naughton SJ, Tarrants JL, Mac Naughton MM, Davis RH (1985) Silica as a defense against herbivory and a growth promotor in African grasses. Ecology 66: 528–535.

[91] Solounias N, Semprebon G (2002) Advances in the reconstruction of ungulates ecomorphology with application to early fossil equids. American Museum Novitates 3366: 1–49.

[92] Merceron G, Kaiser TM, Kostopoulos DS, Schulz E (2010) Ruminant diet and the Miocene extinction of European great apes. Proceedings of the Royal Society B 277: 3105–3112. 20519220 10.1098/rspb.2010.0523 PMC2982054

[93] Walker A, Hoeck HN, Perez L (1978) Microwear of mammalian teeth as an indicator of diet. Science 201: 908–910. 684415 10.1126/science.684415

[94] Ramdarshan A, Alloing-Séguier T, Merceron G, Marivaux L (2012) Ecological niche partitioning in a modern South American primate community: implications for extinct species. PLoS One 6: e27392. 10.1371/journal.pone.0027392 PMC3208638 22076156

[95] Teaford MF, Robinson JG (1989) Seasonal or ecological differences in diet and molar microwear in *Cebus nigrivittatus* . American Journal of Physical Anthropology 80: 391–401. 2686463 10.1002/ajpa.1330800312

[96] Ehleringer JR, Cerling TE, Helliker BR (1997) C_4_ photosynthesis, atmospheric CO_2_, and climate. Oecologia 112: 285–299. 28307475 10.1007/s004420050311

[97] Quade J, Cerling TE, Andrews P, Alpagut A (1995) Paleodietary reconstruction of Miocene faunas from Pasalar, Turkey using stable carbon and oxygen isotopes of fossil tooth enamel. Journal of Human Evolution 28: 373–384.

[98] Passey BH, Cerling TE, Perkins ME, Voorhies MR, Harris JM, et al (2002) Environmental change in the great plains: An isotopic record from fossil horses. The Journal of Geology 110: 123–140.

[99] Van der Merwe NJ, Medina E (1989) Photosynthesis and ^13^C/^12^C ratios in Amazonian rain forests. Geochimica et Cosmochimica Acta 53: 1091–1094.

[100] Yakir D (1997) Oxygen-18 of leaf water: a crossroad for plant associated isotopic signals. In: Griffith H, editor. Stable isotopes: integration of biological, ecological and geochemical processes. Oxford: BIOS. pp. 147–168.

[101] Mihlbachler MC, Solounias N (2006) Coevolution of tooth crown height and diet in Oreodonts (Merycoidodontidae, Artiodactyla) examined with phylogenetically independant contrast. Journal of Mammalian Evolution 13: 11–36.

[102] de Bonis L, Bouvrain G, Geraads D, Koufos GD (1992) Multivariate study of the late Cenozoic mammalian faunal compositions and paleoecology. Paleontologia i Evolució 24–25: 93–101.

[103] Fortelius M, Eronen J, Liu L, Pushkina D, Tesakov A, et al (2006) Late Miocene and Pliocene large land mammals and climatic changes in Eurasia. Palaeogeography, Palaeoclimatology, Palaeoecology 238: 219–227.

[104] Fortelius M, Eronen J, Liu L, Pushkina D, Tesakov A, et al.. (2003) Continental-scale hypsodonty patterns, climatic paleobiogeography and dispersal of Eurasian Neogene large mammal herbivores. In: Reumer JWF, Wessels W, editors. Distribution and Migration of Tertiary Mammals in Eurasia: DEINSEA. pp. 1–11.

[105] Zhang Z-Q, Gentry AW, Kaakinen A, Liu L-P, Lunkka JP, et al (2002) Land mammal faunal sequence in the late Miocene of China: new evidence from Lantian, Shaanxi province. Vertebrata Palasiatica 40: 166–176.

[106] Laden G, Wrangham R (2005) The rise of the hominids as an adaptative shift in fallback foods: Plant underground strorage organs (USOs) and australopith origins. Journal of Human Evolution 49: 482–498. 16085279 10.1016/j.jhevol.2005.05.007

[107] Feranec RS (2003) Stable isotopes, hypsodonty, and the paleodiet of *Hemiauchenia* (Mammalia: Camelidae): a morphological specialization creating ecological generalization. Paleobiology 29: 230–242.

[108] Kahlke R-D, García N, Kostopoulos DS, Lacombat F, Lister AM, et al (2011) Western Palaearctic palaeoenvironmental conditions during the Early and early Middle Pleistocene inferred from large mammal comunities, and implications for hominin dispersal in Europe. Quaternary Science Reviews 30: 1368–1395.

[109] Jernvall J, Fortelius M (2002) Common mammals drive the evolutionary increase of hypsodonty in the Neogene. Nature 417: 538–540. 12037565 10.1038/417538a

[110] Kaiser TM (2009) *Anchitherium aurelianense* (Equidae, Mammalia)–a brachydont “dirty browser” in the community of herbivorous large mammals from Sandelzhausen (Miocene, Germany). Paläontologische Zeitschrift 83: 131–140.

[111] Daxner-Höck G, Bernor RL (2009) The early Vallesian vertebrates of Atzelsdorf (Late Miocene, Austria) 8. *Anchitherium*, Suidae, and Castoridae (Mammalia). Annalen des Naturhistorischen Museums in Wien, Serie A 111: 557–584.

[112] Sondaar PY (1974) The Hipparion of the Rhone valley. Geobios 7: 289–306.

[113] Eronen JT, Evans AR, Fortelius M, Jernvall J (2010) The impact of regional climate on the evolution of mammals: a case study using fossil horses. Evolution 64: 398–408. 19735525 10.1111/j.1558-5646.2009.00830.x

[114] Koch PL, Tuross N, Fogel ML (1997) The effects of sample treatment and diagenesis on the isotopic integrity of carbonate in biogenic hydroxylapatite Journal of Archaeological Science. 24: 417–429.

[115] Spötl C, Vennemann TW (2003) Continuous-flow isotope ratio mass spectrometric analysis of carbonate minerals. Rapid Communication in Mass Spectrometry 17: 1004–1006. 10.1002/rcm.1010 12717777

[116] King T, Andrews P, Boz B (1999) Effect of taphonomic processes on dental microwear. American Journal of Physical Anthropology 108: 359–373. 10096686 10.1002/(SICI)1096-8644(199903)108:3<359::AID-AJPA10>3.0.CO;2-9

[117] Mihlbachler MC, Brian BL, Caldera-Siu A, Chan D, Lee R (2012) Error rates and observer bias in dental microwear analysis using light microscopy. Palaeontologia Electronica 15: 1–22.

[118] Merceron G, Blondel C, de Bonis L, Koufos GD, Viriot L (2005) A new dental microwear analysis: application to extant Primates and *Ouranopithecus macedoniensis* (Late Miocene of Greece). Palaios 20: 551–561.

[119] DeGusta D, Vrba E (2005) Methods for inferring paleohabitats from discrete traits of the bovid postcranial skeleton. Journal of Archaeological Science 32: 1115–1123.

[120] Kaiser TM, Bernor R, Fortelius M, Scott R (2000) Ecological diversity in the Neogene genus *Hippotherium* (Perissodactyla, Equidae) from the late Miocene of Central Europe. Journal of Vertebrate Paleontology 20 Supplement: 51A

[121] Kaiser TM, Fortelius M (2003) Differential mesowear in occluding upper and lower molars: Opening mesowear analysis for lower molars and premolars in hypsodont horses. Journal of Morphology 258: 63–83. 10.1002/jmor.10125 12905535

[122] Kaiser TM (2011) Feeding ecology and niche partitioning of the Laetoli ungulate faunas. In: Harrison T, editor. Paleontology and Geology of Laetoli: Human Evolution in Context: Volume 2: Fossil Hominins and the Associated Fauna (Vertebrate Paleobiology and Paleoanthropology). Springer. pp. 329–354.

[123] Semprebon GM, Rivals F (2007) Was grass more prevalent in the pronghorn past? An assessment of the dietary adaptations of Miocene to Recent Antilocapridae (Mammalia: Artiodactyla). Palaeogeography, Palaeoclimatology, Palaeoecology 253: 332–347.

