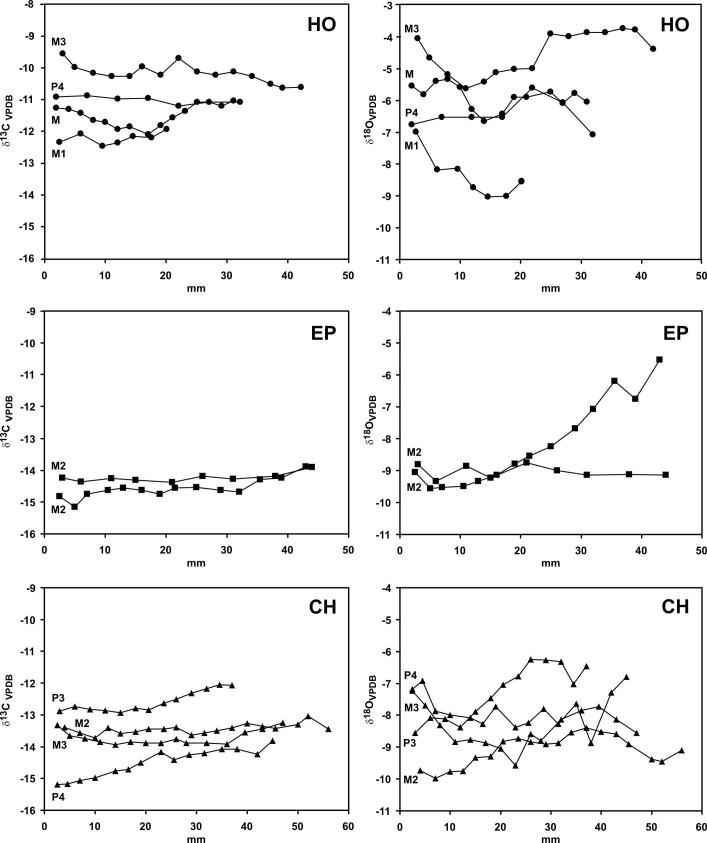# Correction: Opportunistic Feeding Strategy for the Earliest Old World Hypsodont Equids: Evidence from Stable Isotope and Dental Wear Proxies

**DOI:** 10.1371/annotation/03028b14-c992-442b-a6a1-ee3ca4132a33

**Published:** 2013-11-14

**Authors:** Thomas Tütken, Thomas M. Kaiser, Torsten Vennemann, Gildas Merceron

In Figure 3, the y-axis is incorrect and should be the delta sign. Please see the corrected Figure 3 here: 

**Figure:**