# Supplementary figures and images for: Opportunistic Feeding Strategy for the Earliest Old World Hypsodont Equids: Evidence from Stable Isotope and Dental Wear Proxies
Source: PLoS One. 2013 Sep 11;8(9):e74463. doi: 10.1371/journal.pone.0074463 (PMC3770545; doi:10.1371/journal.pone.0074463)

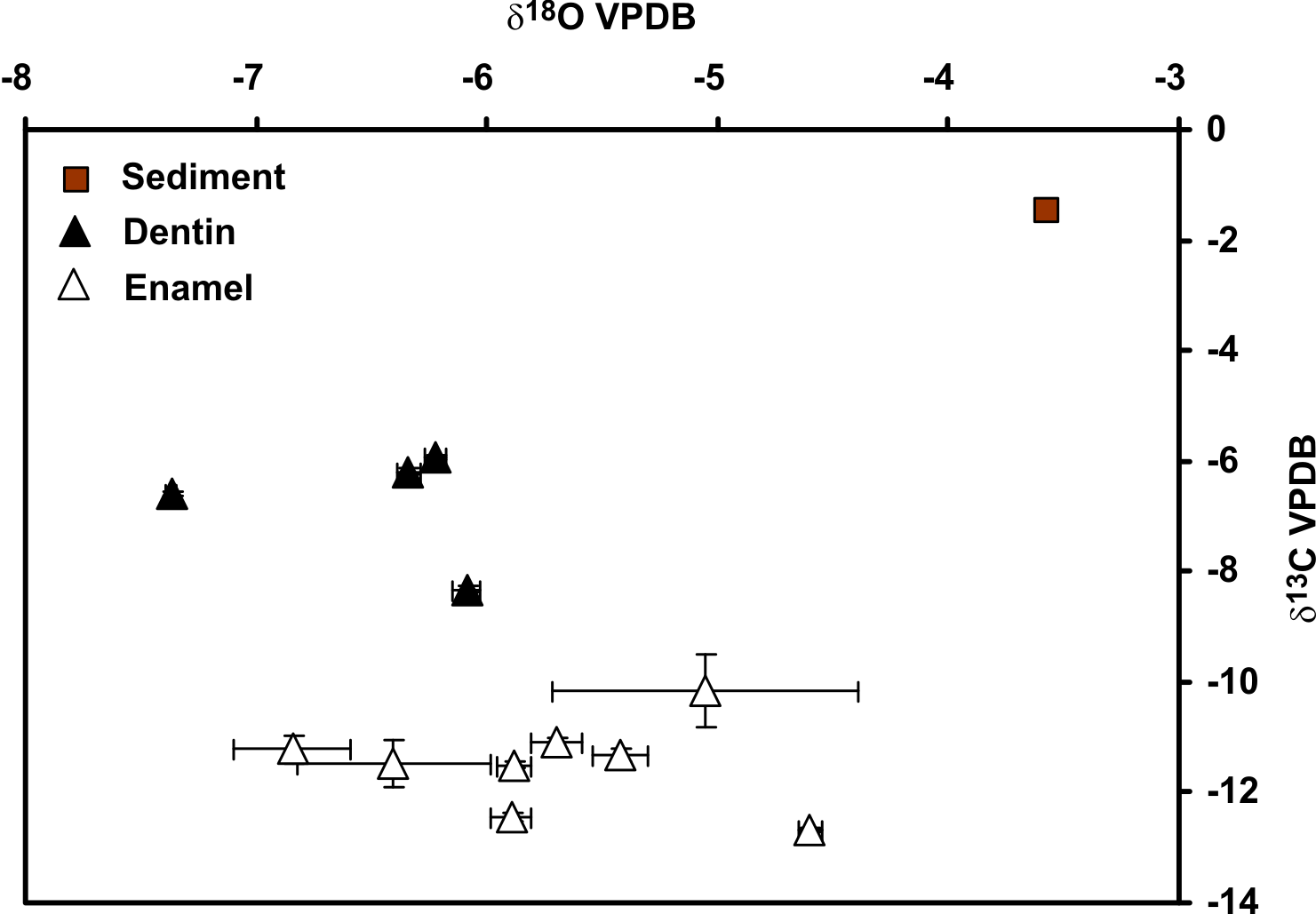

Supplement: Figure S1 — δ13C and δ18O values of enamel and dentin H. primigenium teeth from the Höwenegg locality as well as on sediment sample from the main fossil-bearing layer. Note the large difference in δ13C values indicating a diagenetic alteration of the dentine while enamel still has values typical for C3 feeders. (TIF) [file pone.0074463.s001.tif]
