# Supplementary material for: Opportunistic Feeding Strategy for the Earliest Old World Hypsodont Equids: Evidence from Stable Isotope and Dental Wear Proxies
Source: PLoS One. 2013 Sep 11;8(9):e74463. doi: 10.1371/journal.pone.0074463 (PMC3770545; doi:10.1371/journal.pone.0074463)
Supplement: Table S4 — Summary statistics of the main dental microwear variables for the browsing and grazing ungulates that composed the comparative dataset. (DOC) [file pone.0074463.s005.doc]

**Table S4.** Summary statistics (number of individuals n, mean m and standard deviation sd) of the main dental microwear variables for the browsing and grazing ungulates that composed the comparative dataset (a). Results of the discriminant analysis on the mixed model including extant browsing and grazing ungulates (artiodactyls and perissodactyls) and dental microwear variables.

| (a) |  |  | Ls* | | Ns | | Nws | | Np | | Nlp | | Pp | | Tot | |
| --- | --- | --- | --- | --- | --- | --- | --- | --- | --- | --- | --- | --- | --- | --- | --- | --- |
| Diet | Species | n | m | sd | m | sd | m | sd | m | sd | m | sd | m | sd | m | sd |
| Browsers | *Capreolus capreolus* | 128 | 126.7 | 34.3 | 21.8 | 6.5 | 1.0 | 1.0 | 32.6 | 12.5 | 4.8 | 2.6 | 58.8 | 10.9 | 54.4 | 14.5 |
|  | *Cephalophus dorsalis* | 19 | 135.3 | 22.6 | 27.2 | 9.3 | 3.0 | 2.1 | 45.6 | 17.4 | 7.9 | 4.4 | 61.6 | 14.3 | 72.7 | 17.4 |
|  | *Cephalophus sylvicultor* | 8 | 183.9 | 52.4 | 19.9 | 4.5 | 1.4 | 1.8 | 34.6 | 13.4 | 5.6 | 4.1 | 62.3 | 9.0 | 54.5 | 15.4 |
|  | *Diceros bicornis* | 10 | 189.1 | 36.2 | 17.7 | 5.9 | 1.2 | 1.6 | 39.5 | 19.1 | 6.1 | 3.5 | 66.0 | 15.2 | 57.2 | 20.4 |
|  | *Giraffa camelopardalis* | 16 | 157.5 | 44.9 | 15.1 | 5.1 | 0.8 | 1.1 | 29.3 | 14.6 | 4.0 | 3.0 | 64.0 | 13.7 | 44.4 | 15.0 |
|  | *Litocranius walleri* | 16 | 141.8 | 47.2 | 15.1 | 2.3 | 0.9 | 0.8 | 34.5 | 19.5 | 6.8 | 5.8 | 66.6 | 9.5 | 49.6 | 20.2 |
|  | *Muntiacus muntjak* | 8 | 139.7 | 31.0 | 28.0 | 7.7 | 0.8 | 0.9 | 51.1 | 11.1 | 4.0 | 2.1 | 64.4 | 9.9 | 79.1 | 10.3 |
|  | *Odocoileus virginianus* | 11 | 126.0 | 35.0 | 19.9 | 5.8 | 1.2 | 1.0 | 31.1 | 10.8 | 5.8 | 5.0 | 60.1 | 13.3 | 51.0 | 11.1 |
|  | *Sylvicapra grimmia* | 24 | 156.5 | 36.5 | 20.9 | 5.8 | 1.1 | 1.2 | 39.1 | 15.1 | 6.0 | 3.7 | 63.5 | 11.8 | 60.1 | 14.5 |
|  | *Tapirus bairdii* | 2 | 213.2 | 12.1 | 20.5 | 12.0 | 2.0 | 1.4 | 25.0 | 12.7 | 3.0 | 2.8 | 54.7 | 27.1 | 45.5 | 0.7 |
|  | *Tapirus terrestris* | 5 | 174.8 | 33.6 | 19.6 | 5.0 | 1.0 | 0.7 | 49.4 | 12.9 | 6.0 | 4.9 | 71.1 | 6.4 | 69.0 | 14.7 |
|  | *Tragelaphus strepsiceros* | 8 | 137.8 | 11.3 | 14.9 | 4.2 | 0.6 | 0.7 | 31.9 | 12.7 | 4.5 | 2.3 | 66.6 | 10.9 | 46.8 | 13.5 |
| Grazers | *Alcelaphus buselaphus* | 36 | 154.0 | 39.6 | 28.0 | 5.8 | 1.9 | 1.8 | 17.8 | 8.3 | 3.1 | 2.6 | 37.4 | 9.2 | 45.8 | 12.2 |
|  | *Damaliscus lunatus* | 14 | 137.2 | 32.3 | 22.6 | 5.3 | 1.5 | 1.5 | 12.7 | 7.1 | 3.9 | 2.1 | 33.7 | 9.8 | 35.4 | 11.6 |
|  | *Equus quagga* | 24 | 196.1 | 35.0 | 22.7 | 4.7 | 2.9 | 1.7 | 12.0 | 8.0 | 3.1 | 2.9 | 32.1 | 12.5 | 34.8 | 10.1 |
|  | *Equus przewalskii* | 6 | 152.4 | 34.0 | 29.8 | 3.7 | 0.7 | 0.8 | 11.7 | 5.1 | 2.2 | 1.5 | 27.2 | 6.6 | 41.5 | 8.0 |
|  | *Hippotragus niger* | 13 | 175.3 | 29.9 | 27.7 | 3.9 | 2.8 | 1.5 | 14.2 | 7.2 | 4.0 | 3.5 | 32.3 | 10.5 | 41.9 | 9.6 |
|  | *Kobus kob* | 26 | 177.9 | 41.5 | 26.3 | 4.6 | 2.7 | 1.6 | 16.9 | 7.8 | 3.9 | 2.5 | 37.4 | 11.7 | 43.2 | 9.8 |
|  | *Ourebia ourebi* | 18 | 173.5 | 21.3 | 22.8 | 4.6 | 1.8 | 1.6 | 20.9 | 13.0 | 2.8 | 2.0 | 44.2 | 17.7 | 43.6 | 12.7 |
|  | *Syncerus caffer* | 24 | 180.8 | 46.5 | 24.6 | 6.6 | 2.6 | 1.5 | 13.3 | 6.5 | 3.7 | 1.7 | 33.8 | 9.3 | 37.8 | 11.7 |

* Ls: lenght of scratches; Ns: number of scratches; Nws: number of wide scratches; Np: number of pits; Nlp: number of large pits;

| *(b) Prior probabilities of groups* | | | | | |  | | | | | | | | | |
| --- | --- | --- | --- | --- | --- | --- | --- | --- | --- | --- | --- | --- | --- | --- | --- |
| Groups | p (%) | | | | |  | | | | | | | | | |
| Browsers | 61.30 | | | | |  | | | | | | | | | |
| Grazers | 38.70 | | | | |  | | | | | | | | | |
|  | | | | | |  | | | | | | |  | | |
| *Misclassification rates* | | | | | | Misclassification (%) | | | | | | |  | | |
| Set of variables | | | | | | Total | | Browsers | | Grazers | | |  | | |
| Ns, Np, Ls, Pp, Tot, Nws, Nfs, Nlp, Nsp | | | | | | 11.54 | | 5.88 | | 20.49 | | |  | | |
|  | | | | | | | | | | | | |  | | |
| *Coefficients* ***a*** *of linear discriminants* | | | | | |  | | | | | | | | |  |
| Variables | Ns | | Nws | | Np | Ngp | Pp | | Tot | | Ls | Nsp | | Nfs | |
| ***a*** | 3.850 | | -0.427 | | -38.453 | 0.165 | 31.48 | | 40.510 | | 0.603 | -0.980 | | -5.550 | |
|  |  | |  | |  |  |  | |  | |  |  | |  | |
| Non-parametric Mann-Whitney test, browsers versus grazers. | | | | | | | | | | | | | | | |
| Variable | | *U* | |  | *p* |  |  | |  | |  |  | |  | |
| LD1 | | 2345.0 | |  | < 0.05 |  |  | |  | |  |  | |  | |
