# Supplementary material for: Opportunistic Feeding Strategy for the Earliest Old World Hypsodont Equids: Evidence from Stable Isotope and Dental Wear Proxies
Source: PLoS One. 2013 Sep 11;8(9):e74463. doi: 10.1371/journal.pone.0074463 (PMC3770545; doi:10.1371/journal.pone.0074463)
Supplement: Table S5 — List of extant species used for mesowear analysis. (DOC) [file pone.0074463.s006.doc]

## Table S5. List of extant species used for mesowear analysis after the database published by Fortelius and Solounias [89]. Only the medium-sized species.

**Browsers:**

AA = *Alces alces*, AM = *Antilocapra americana*, DB = *Diceros bicornis*, CC = *Capreolus capreolus*, GC = *Giraffa camelopardalis*, LW = *Litocranius walleri*, OH = *Odocoileus hemionus*, OV = *Odocoileus virginianus*, TE = *Tragelaphus eurycerus*, TS = *Tragelaphus scriptus.*

**Mixed feeders:**

AP = *Axis porcinus*, AX = *Axis axis*, BT = *Budorcas taxicolor*, CD = *Cervus duvaucelii*, CA = *Capricornis sumatraensis*, CI = *Capra ibex*, CL = *Camelus dromedarius*, CC = *Cervus canadensis*, CU = *Cervus unicolor*, GG = *Gazella granti*, GT = *Gazella thomsoni,* LG = *Lama glama*, LV = *Lama vicugna*, MA = *Antidorcas marsupialis*, ME = *Aepyceros melampus*, OC = *Ovis canadensis*, OM = *Ovibos moschatus*, TA = *Tragelaphus angasi*, TI = *Tragelaphus imberbis*, TO = *Taurotragus oryx*, TQ = *Tetracerus quadricornis*, TR = *Buselaphus tragocamelus*.

**Grazers:**

AB = *Alcelaphus buselaphus*, AL = *Alcelaphus lichtensteinii*, BB = *Bison bison*, CS = *Ceratotherium simum*, CT = *Connochaetes taurinus*, EB = *Equus quagga*, EG = *Equus grevyi*, HE = *Hippotragus equinus*, HN= *Hippotragus niger*, KE = *Kobus ellipsiprymnus*, OO = *Ourebia ourebi*, RR = *Redunca redunca,* SC *= Syncerus caffer*.

## Fruit-eaters (=mabra group after Fortelius and Solounias [89]): DR = Cephalophus dorsalis, NG = Cephalophus nigrifrons, NI = Cephalophus niger, SL = Cephalophus sylvicultor, HY = Hyaemoschus aquaticus.
