# Supplementary material for: Opportunistic Feeding Strategy for the Earliest Old World Hypsodont Equids: Evidence from Stable Isotope and Dental Wear Proxies
Source: PLoS One. 2013 Sep 11;8(9):e74463. doi: 10.1371/journal.pone.0074463 (PMC3770545; doi:10.1371/journal.pone.0074463)
Supplement: Text S1 — Detailed site description of the fossil localites. (DOC) [file pone.0074463.s007.doc]

**Text S1: Supporting information**

**The *Hippotherium primigenium* populations and their palaeoenvironment**

### *The Eppelsheim locality -* Deposits of the Miocene Rhine River are exposed at many places in the Rheinhessen area, Germany. This late Miocene sequence of sands, silts, gravels and conglomerates, which have been extensively quarried during the 19^th^ and early 20^th^ century, yielded a rich mammalian fauna, which mostly consists of disarticulated large mammal remains. The deposits are known as the Dinotheriensande, named after the abundant deinothere remains and were recently defined as Eppelsheim Formation . The Dinotheriensande localities are all stratigraphically placed within the lower part of MN9, with an estimated age of about 10.5 Ma . In addition to Late Miocene (Vallesian) taxa the Dinotheriensand Fauna also comprises early and late Middle Miocene mammal faunas . The Dinotheriensande complex is a fluvial deposit along the old course of the Rhine River. Mammalian fossils are almost entirely restricted to a sand/gravel-dominated horizon close to the base of the sequence . Bartz reconstructed a SE-NW direction for the former course of the Dinotheriensande river system responsible for the deposition of the fossiliferous sediments in a high-energy depositional environment . Due to the lack of pollen the reconstruction of the vegetation in the river basin is given only by plant macro remains from a leaf flora of the Dinotheriensande locality of Sprendlingen . This flora consists of roughly 1000 leaf specimens of *Ulmus*, *Salix*, Betulaceae, *Zelkova*, *Quercus*, *Taxodium*, *Acer, Populus* and *Fagus*, which represent a fraction of the most abundant members of a mixed mesophytic forest flora . Meller concludes that this flora represents a typical Neogene meadow forest of the *Ulmus*-*Salix*-type.

The chronological homogeneity of the Dinotheriensande assemblage is uncertain. However, this study focused on Eppelsheim (EP), situated 30 km south of the city of Mainz, which has yielded the richest assemblage of mammalian remains among the Dinotheriensande complex . Additionally this assemblage comprises the type material of *Hippotherium primigenium* (MEYER, 1829), which now is housed in the collections of the Forschungsinstitut and Naturmuseum Senckenberg (Frankfurt).

### *The Höwenegg locality -* Höwenegg is the northern-most volcano of the Tertiary Hegau volcanic field, southwestern Germany. Close to this volcano fossiliferous marly limnic deposits and reworked tuffs, so-called “Höwenegg-Schichten”, of a shallow, late Miocene freshwater lake, are intercalated between two tuff layers an older “Hornblende Tuff” and an overlying “Jüngerer Tuff”. The “Höwenegg-Schichten” constitute the Höwenegg Formation that was deposited in a lake that formed after the eruption of the older hornblende pyroclastics in the late Miocene . Lake sedimentation lasted at most until the eruption of the younger tuff and the basalt forming the Höwenegg volcano. Radiometric dating suggests an age of 7-6 Ma for the Höwenegg basalt and 12.4±1 Ma for a hornblende tuff from the Höwenegg profile . The Höwenegg Formation itself was recently radiometrically dated to 10.3±0.19 Ma by means of a single hornblende crystal ^40^Ar/^39^Ar age from the hornblende tuff of the Höwenegg sequence .

The Höwenegg Formation is famous for the preservation of complete articulated mammal skeletons from various species of Artiodactyla and Perissodactyla, especially the hipparionine *Hippotherium primigenium* . The occurence of this moderately high-crowned equid places the Höwenegg Formation into MN9 and is one of the first occurences of this species in Europe (“*Hipparion* Datum”) . According to sedimentological investigations, the vertebrate remains were probably accumulated in the sediments of a shallow anaerobic lake and have been deposited within a short time interval . The mammal remains are found in association with an invertebrate fauna of gastropods, ostracods as well as plant fossils embedded in marly lake sediments . Plant remains are relatively abundant, however, they represent only a little diverse taphocenosis, which shows a high correspondence with other Central European middle and late Miocene localities . The flora, which is dominated by the mediterranean genus *Celtis,* is a deciduous flora, indicating a warm-temperate character, indicative for warm mesophytic forests with little seasonality . This is in accordance with the palaeoecological and palaeoenviromental interpretations based on the mammalian fauna and stable isotope compositions of mammal teeth .

*The Soblay locality -* The late Miocene deposits in Soblay (Ain, France) are composed of a several meters thick sequence of lignites alternating with marls, which overly Upper Jurassic calcareous marine sediments. The fossiliferous sediments were deposited in a doline. The late Miocene lignites and marls have been quarried during the 19^th^ and 20^th^ centuries. Since 1944, the exact location of the fossil remains within the stratigraphic sequence is known. They all belong to the second lignite unit that has yielded a rich mammalian fauna including 67 species of large and small mammals . The latter are precious as they can correlate the fauna with other late Miocene European localities. Based on old collections Guérin and Mein and on new findings thanks to Ménouret and Mein , Soblay is biostratigraphically correlated to the European mammal reference zone MN10. Indeed, several taxa of rodents occurred only during the Vallesian mammalian stage, more specifically during the late Vallesian. Among them, there are the cricetine *Rotundomys bressanus* and the murine *Progonomys* cf. *cathalai*. Also, the presence of *Hippotherium* rejects a pre-Vallesian age. It is worth to note that one of the last occurrences of *Anchitherium* in Eurasia is known from Soblay, documented by an astragalus.

The environment of Soblay is depicted as a wet, forested area. Indeed, the abundance of glirids (6 species) and especially the co-occurrence of four species of flying squirrels (*Blackia miocaenica*, *Albanensia grimmi*, *Hylopetes* sp. and *Pliopetaurista bressana*) indicate the presence of dense forested habitats in Soblay. Besides, the co-occurence of two castorids (*Chalicomys jaegeri* and *Trogontherium minutum*) and the presence of *Diplocynodon*, a small-sized crocodile, indicate wet and warm conditions during the late Vallesian in the region of Soblay . Furthermore, Jiménez-Moreno reports the abundance of pollen diagnostic for arboreal species that favour warm and even subtropical climatic conditions. Xeric and steppe elements represent less than 5% of the pollinic spectrum, while about 35% of the pollen assemblage is composed of grasses, forbs, and few shrub-sized dicots. Based on all these faunistic and floristic elements, Ménouret and Mein conclude that Soblay was likely situated in a forested area close to open patches surrounding a water body with prevailing humid and warm climatic conditions.

*The Charmoille locality -* This site is a sand pit close to the small village of Charmoille in the Canton Jura in northern Switzerland. The local abundance of sand and gravels suggests fluvial deposits that are composed of three members of the "Bois de Raube" Formation ("Montchaibeux", "Bois de Raube", and "Ajoie" members). Charmoille belongs to the upper member ("Ajoie"). The mineral composition of the clay fraction is interpreted to have formed by weathering processes under humid subtropical conditions with moderate seasonal changes . The faunal assemblage of Charmoille supports an early Vallesian age . Indeed, the presence of *Hippotherium primigenium* together with three rhinocerotids *Aceratherium incisivum*, *Lartetotherium sansaniensis* and *Dihoplus schleiermacheri* indicate that the locality belongs to the European mammal reference zone MN9 . Several other taxa of Artiodactyla (*Miotragoceros pannoniae, Dorcatherium naui, Euprox dicranocerus, Korynochoerus palaeochoerus, Conohyus simorrensis*), Perissodactyla (*Tapirus priscus*, *Chalicotherium goldfussi*), Proboscidea (*Deinotherium giganteum*, *Tetralophodon longirostris*) and Carnivora (*Machairodus aphanistus*, *Agnotherium* cf. *antiquum*) complete the guild of large mammals . Liniger also describes a community of molluscs from Charmoille with the dominance of taxa indicative of wooded and bushy environments (*Hemicycla, Cepaea*, *Zonites*) with some water-dependent taxa as well (*Unio* and *Planorbis*). This author also describes horizons with leaves attributed to the genus *Populus* (Salicaceae), which today is often associated with the presence of water.

1. Tobien H (1983) Über Hipparion-Reste aus der obermiozänen Süsswassermolasse Südwestdeutschlands. Zeitschrift der Deutschen Geologischen Gesellschaft 90: 178-192.

2. Tobien H (1980) Taxonomic status of some Cenozoic mammalian local faunas from the Mainz basin. Mainzer geowissenschaftliche Mitteilungen 9: 203-235.

3. Grimm MC (2005) Beiträge zur Lithostratigraphie des Pala¨ogens und Neogens im Oberrheingebiet Oberrheingraben, Mainzer Becken, Hanauer Becken). Geologisches Jahrbuch Hessen 132: 79-112.

4. Franzen JL (2011) Eppelsheim Formation. In: Grimm KI, editor. Stratigraphie von Deutschland IX Tertiär, Teil 1: Oberrheingraben und benachbarte Tertiärgebiete. Hannover: Deutsche Gesellschaft für Geowissenschaften. pp. 184-187.

5. Woodburne MO, Bernor RL, Swisher CCI (1996) An appraisal of the stratigraphic and phylogenetic bases for the ”Hipparion Datum” in the Old World. In: Bernor RL, Fahlbusch V, Mittmann HW, editors. The Evolution of Western Eurasian Neogene Mammal Faunas. New York: Columbia University Press. pp. 124-136.

6. Steininger FF (1999) Chronostratigraphy, Geochronology and Biochronology of the Miocene "European Land Mamal Mega-Zones (ELMMZ)" and the Miocene "Mammal-Zones (MN-Zones)". In: Rössner GE, Heissig K, editors. Land Mammals of Europe. München: Verlag Friedrich Pfeil. pp. 9-24.

7. Andrews PA, Bernor RL (1999) Vicariance Biogeography and Paleoecology of Eurasian Miocene hominoid Primates. In: Agusti J, Rook L, Andrews P, editors. The Evolution of Neogene Terrestrial Ecosystems in Europe. Cambridge: Cambridge University Press, . pp. 454-488.

8. Böhme M, Aiglstorfer M, Uhl D, Kullmer O (2012) The Antiquity of the Rhine River: Stratigraphic Coverage of the Dinotheriensande (Eppelsheim Formation) of the Mainz Basin (Germany). PLoS ONE 7: e36817.

9. Tobien H (1983) Bemerkungen zur Taphonomie der spättertiären Säugerfauna aus den Dinotheriensanden Rheinhessens (Bundesrepublik Deutschland). Weltenburger Akademie 1983: 191-200.

10. Bartz J (1936) Das Unterpliozän in Rheinhessen. Jahresberichte und Mitteilungen des oberrheinischen geologischen Vereins NF 2: 121-228.

11. Meller B (1989) Eine Blatt-Flora aus den obermiozänen Dinotheriensanden (Vallesium) von Sprendlingen (Rheinhessen). Documenta naturae 54: 1-109.

12. Mai DH (1981) Entwicklung und klimatische Differenzierung der Laubwaldflora Mitteleuropas im Tertiär. Flora 171: 525-582.

13. Franzen JL, Fejfar O, Storch G, Wilde V, editors (2003) Eppelsheim 2000 - new discoveries at a classic locality. Rotterdam: Deinsea. 217-234 p.

14. Franzen JL (2000) Auf dem Grunde des Urrheins – Ausgrabungen bei Eppelsheim. Natur und Museum 130: 169-180.

15. Schreiner A (1976) Hegau und westlicher Bodensee. Sammlung geologischer Führer 62: 1-93.

16. Lippolt HJ, Gentner W, Wimmenauer W (1963) Altersbestimmungen nach der Kalium-Argon-Methode an tertiären Eruptivgesteinen Südwestdeutschlands Jahreshefte des geologischen Landesamtes in Baden-Württemberg 6.

17. Swisher CC (1996) New 40Ar/39Ar dates and their contribution toward a revised chronology for the late Miocene of Europe and West Asia. In: Bernor RL, Fahlbusch V, Mittmann H-W, editors. The evolution of western Eurasian Neogene mammal faunas. New York: Columbia University Press. pp. 64-77.

18. Munk W, Bernor RL, Heizmann EPJ, Mittmann HW (2007) Excavations at the late Miocene MN9 (10.3 Ma) locality of Höwenegg (Hegau), southwest Germany, 2004-2006. Carolinea 65: 5-13.

19. Tobien H (1986) Die jungtertiaere Fossilgrabungsstaette Höwenegg in Hegau (Südwestdeutschland). Ein Statusbericht. Carolinea 44: 9-34.

20. Bernor RL, Tobien H, Hayek LA, Mittmann HW (1997) *Hippotherium primigenium* (Equidae, Mammalia) from the late Miocene of Höwenegg (Hegau, Germany). Andrias 10: 1-230.

21. Bernor RL, Koufos GD, Woodburne MO, Fortelius M (1996) The evolutionary history and biochronology of European and Southwest Asian Late Miocene and Pliocene Hipparionine Horses. In: Bernor RL, Fahlbusch V, Mittmann H-W, editors. The evolution of Western Eurasian Neogene mammal faunas. New York: Columbia University Press. pp. 307-338.

22. Woodburne MO, Theobald G, Bernor RL, Swisher CCI, König H, et al. (1996) Advances in Geology and Stratigraphy at Höwenegg, South western Germany. In: R.L. B, Fahlbusch V, Mittmann HW, editors. The Evolution of Western Eurasian Neogene Mammal Faunas. New York: Columbia University Press. pp. 106-123.

23. Gregor HJ (1982) Die jungtertiären Floren Süddeutschlands. Paläokarpologie, Phytostratigraphie, Paläoökologie, Paläoklimatologie. Stuttgart: Ferdinand Enke. 278 p.

24. Kirchheimer F (1957) Die Laubgewächse der Braunkohlenzeit. Halle/Saale: Knapp. 78 p.

25. Tütken T, Poppe H (2011) Paleoenvironment and habitat of Late Miocene mammals from Höwenegg, SW Germany inferred from O, C and Sr isotope compositions of fossil teeth. Geological Society of America Bulletin 43: 212.

26. Ménouret B, Mein P (2008) Les vertébrés du Miocène supérieur de Soblay (Ain, France). Documents du Laboratoire de Géologie de Lyon 165: 1-97.

27. Guerin C, Mein P (1971) Les principaux gisements de mammifères miocènes et pliocènes du domaine rhodanien. Docum Lab Geol Univ Lyon H.S.: 131-170.

28. Jiménez-Moreno G (2005) Utilización del análisis polínico para la reconstrucción de la vegetación, clima y estimacion de paleolaltitudes a lo largo del arco alpino durante el Mioceno (21-8 Ma). Grenada: University of Grenada.

29. Becker D (2003) Paléoécologie et paléoclimats de la Molasse du Jura (Oligo-Miocène): apport des Rhinocerotoidea (Mammalia) et des minéraux argileux. GeoFocus 9: 327.

30. Kälin D (1997) Litho- und Biostratigraphie der mittel- bis obermiozänen Bois de Raube-Formation (Nordwestschweiz). Eclogae Geologicae Helvetiae 90: 97-114.

31. Schäfer H (1961) Die pontische Säugetierfauna von Charmoille (Jura bernois). Eclogae geologicae Helvetiae 54: 559-566.

32. Liniger H (1925) Geologie des Delsberger Beckens un der Umgebung von Movelier. Beiträge Geologischen Karte der Schweiz NF 55: 1-71.
